# Integrating Emotional Contagion into Leadership Theorizing: Development and Validation of the Leader Awareness of Holistic Contagion Scale

**DOI:** 10.3390/ejihpe16050061

**Published:** 2026-04-29

**Authors:** Laura Petitta, Lixin Jiang

**Affiliations:** 1 Department of Psychology, Sapienza University of Rome, Via dei Marsi, 78, 00185 Rome, Italy; 2 School of Psychology, University of Auckland, 23 Symonds Street, Auckland 1010, New Zealand; l.jiang@auckland.ac.nz

**Keywords:** emotional contagion, leadership, new contagion leadership scale, cross-country, follower outcomes

## Abstract

While the literature acknowledges the importance of emotion management for effective leadership, no leadership theory embeds the management of contextual emotions that involuntarily spread among multiple workplace stakeholders (i.e., holistic emotional contagion) and are jointly intertwined with leaders’ actions. The present research aimed to: (1) include emotional contagion into leadership theorizing and assess the cross-country validity of the accompanying measure (Leader Awareness of Holistic Contagion Scale; LAHCS), and (2) examine the LAHCS’ convergent, discriminant and nomological/criterion validity. Data (Study 1) from 1454 Italian employees supported the LAHCS construct and convergent validity with multiple leadership scales and discriminant validity against group-member-prototypicality. Data (Study 2) from the U.S. (*N* = 400) and Italy (*N* = 186) supported measurement invariance. SEM model results suggest that leaders’ awareness of holistic contagion and their orientation to manage contagion are associated with higher followers’ commitment and leadership satisfaction. Interestingly, the leader’s engagement in active exploration of contagion exchanges and their awareness of the leader–follower emotional distance is associated with followers’ higher burnout, lower commitment and leadership dissatisfaction. In conclusion, our cross-country findings support the LAHCS validity and reveal that leaders who are aware of workplace emotional traffic are appreciated. Notably, if they attempt to actively explore this traffic or are aware of followers’ emotional distance, then the situation becomes likely intrusive and uncomfortable, resulting in followers’ dissatisfaction, poor commitment and distress. For scholars and practitioners alike, our findings provide a leadership conceptual framework, including emotional contagion as a springboard to the understanding of some apparently inconvenient truths about emotions and leadership.

## 1. Introduction

A converging perspective on leadership tends to see leaders as individuals who use their influence to channel followers’ efforts to achieve organizational goals (Yukl & van Fleet, 1992) and as key agents who utilize both technical (e.g., strategizing) and social (e.g., influencing) skills to ensure followers function effectively (Jex & Britt, 2008). Relatedly, the use of emotions in leadership processes is considered a necessary component for overcoming obstacles and performing well (e.g., Halimah et al., 2014). Therefore, the efficacy of the leader’s influence to steer followers’ efforts to achieve common goals may be enhanced by their understanding of the emotions of individuals and the effects they have on their behavior.

While most of the existing leadership literature has extensively acknowledged the key role of emotions and how leaders use them in order to enhance effective leadership and achieve success (e.g., Antonopoulou, 2024; Halimah et al., 2014), few leadership theories (e.g., servant and transformational) embed emotion management within their theorizing and accompanying operationalization. Moreover, no theory does it from the perspective of the leader’s mastery of contextual emotions that involuntarily spread among multiple stakeholders at work (i.e., holistic emotional contagion; Hatfield et al., 1993; Petitta & Naughton, 2015). Briefly, the term “holistic” refers to the investigation of the contagion exchanges far off the typical dyadic (i.e., two distinct roles) organizational relationships, such as emotional exchanges only between leader and follower or between employee and customer, and designates the study of the multiple transfers of emotions simultaneously occurring among leaders, coworkers, and clients (i.e., all the primary organizational stakeholders) due to their daily working interactions. Indeed, the contagion perspective stands out as opposed to more common person-centered emotional processes such as a leader’s emotional intelligence (i.e., sensitivity towards and efficacious use of one’s and others’ emotions; Goleman, 1995) or empathy (i.e., understanding others’ emotions and willingness to consider them in one’s own actions; N. Kock et al., 2019).

The present paper engages an inter-personal approach to examining emotions and leadership and aims at complementing existing leadership models by proposing a conceptual framework that embeds leaders’ mastery of emotional contagion dynamics or rather, contextualizes emotional exchanges within a broader “emotional orbit” including multiple stakeholders at work (e.g., leaders, followers, clients) beyond the leader–follower(s) dyad. Specifically, the goal of the research was threefold.

First, we seek to include emotional contagion (i.e., the involuntary absorption of others’ emotions during social encounters; Hatfield et al., 1994) into leadership theorizing and thus provide a new holistic and multi-foci (i.e., different sources of absorbed emotions) emotional leadership model (i.e., the Leader Awareness of Holistic Contagion). This model refers to leaders’ awareness of and willingness to manage holistic emotional exchanges among different roles (i.e., multiple stakeholders such as leaders, colleagues and clients) in their own context. Indeed, the literature highlights the relevance of leaders’ emotional intelligence (e.g., Lubbadeh, 2020) or empathy (e.g., Muss et al., 2025) for effective leadership action. Notably, both these constructs focus on within-person emotional processes within the leader or the followers. Moreover, they refer to separate emotion-related mechanisms not intrinsically embedded in leadership models. The newly developed contagion leadership model shifts the focus towards contextual dynamics of emotional exchanges (i.e., an *inter-personal* perspective) occurring between a leader and followers, but also inevitably occurring with other factual stakeholders within the workplace with whom members interact (e.g., clients or externals to the organization). In so doing, we extend the literature on the emotion–leadership link by incorporating emotion management into leadership theorizing and proposing a framework embedding leaders’ mastery of an “emotional traffic” occurring in their workplace that expands emotion management beyond the leader–follower(s) dyad (i.e., not just between the leader and their follower(s)).

Second, we aim to develop and examine the psychometric properties of a new leadership scale rooted in the contagion management model—the Leader Awareness of Holistic Contagion Scale. The tool was developed within the new contagion leadership model. This tool can be used to assist leaders in becoming mindful of their emotional exchanges within a broader emotional setting than that of the leader–follower dyadic relationship, while improving understanding of their own and others’ emotions (Yukl, 2012) that emerge from complex workplace exchanges and strengthening the leader–member connection. Towards this end, we utilized a multi-sample and cross-country (i.e., U.S., Italy) approach to test the multiple types of validity (e.g., Hinkin, 1998) of the new contagion leadership model scale. Specifically, the research assessed construct, convergent, and discriminant validity (Study 1) using multiple leadership models (i.e., transformational, transactional, servant, authentic, LMX and leader group prototypicality) and a non-leadership construct capturing a social mechanism different from the leadership process (i.e., group member prototypicality; Steffens et al., 2021). Furthermore, we tested measurement invariance, nomological/criterion, and incremental validity of the new scale’s dimensions in predicting followers’ well-being, satisfaction with supervisor and organizational commitment (Study 2). In so doing, we draw our conclusions on independent samples and different cultural contexts that provide ecological validity of our findings and support the applicability of the new scale in organizations from different national contexts because members of different cultures interpret the measure in a conceptually similar way (Deffner et al., 2022).

Third, the current research aimed to examine the extent to which the new contagion leadership model predicts (i.e., nomological/criterion validity; F. Kock et al., 2024) employees’ well-being (i.e., burnout) and work attitudes (i.e., supervisor satisfaction and organizational commitment). Indeed, the literature suggests that empathetic leadership facilitates the understanding of a follower’s work situation and provides emotional security for the followers, thus bearing emotional support that increases their satisfaction and ultimately improves their performance and the overall leader–member connection (N. Kock et al., 2019; Yukl, 2012). Arguably, the new inter-personal and context-focused contagion leadership model that switches from dispositional approaches to emotions (e.g., empathy) towards contextual emotion-related factors as proposed by emotional contagion dynamics may assist in further expanding the understanding of the emotional ambience in the workplace and improving the quality of leader–member interactions that may ultimately promote employees’ well-being and productivity.

Overall, the present research aims at filling the leadership literature’s shortcomings in explicitly embedding emotion management within leadership measurement and conceptualization, as well as the gaps in leadership research that more commonly focus on person-centered emotional processes (e.g., leaders’ emotional intelligence). Consistently, we aimed at complementing extant leadership models and research on leadership and emotions by (1) including workplace emotional contagion (i.e., the involuntary absorption of emotions from multiple stakeholders at work and beyond the leader–follower dyad) into leadership theorizing, thus providing a new holistic and multi-foci (i.e., different sources of absorbed emotions) emotional leadership model (i.e., the Leader Awareness of Holistic Contagion); (2) developing and examining the psychometric properties of a new leadership scale rooted in the contagion management model—the Leader Awareness of Holistic Contagion Scale; and (3) examining the extent to which the new contagion leadership model predicts (i.e., nomological/criterion validity) employees’ well-being (i.e., burnout) and work attitudes (i.e., supervisor satisfaction, organizational commitment).

We thereby contribute to the literature on leadership, and leadership and emotions, by providing a conceptual framework, and accompanying scale, which (a) exclusively focuses on leader’s management of workplace emotional dynamics (rather than tasks), (b) includes emotional dynamics within leadership theorization, rather than examining such dynamics as additional factors external to leadership, as is the case of leader’s attributes such as their emotional intelligence or empathy, (c) focuses on holistic emotional contagion at work (i.e., contextual emotions) or rather, the involuntarily spreading of emotions among multiple stakeholders that surround the leader–member dyad, thus going beyond the more common person-centered emotional processes (e.g., emotional intelligence) in the study of the link between leadership and emotions, and (d) shifts the focus towards contextual dynamics of emotional exchanges (i.e., an *inter-personal* perspective) occurring between a leader and followers as opposed to more common processes from inside the leader or the followers (i.e., an *intra-individual* perspective), such as empathy and emotional intelligence. Moreover, we add to the literature by examining how the newly developed conceptual facets of the LAHC model, or rather, the extent to which leaders deal with emotion-related dynamics and manage contextual emotions at work associated with employees’ organizational attitudes and well-being (e.g., commitment, burnout). From a practical standpoint, our findings can provide scholars and practitioners with an overarching conceptual framework emphasizing that leaders and followers are not isolated and leaders’ actions are embedded in an emotional ambience that is co-created by multiple stakeholders with whom they simultaneously interact and exchange emotions, while also providing a tool to increase leaders’ awareness and mastery of such contagion dynamics.

Below, we initially provide an overview of the theoretical foundations we draw upon for the different leadership models included in the study. We then present the theoretical background for emotional contagion dynamics in the workplace and the literature underpinning our holistic contagion approach to leadership that supports the development of the new Leader Awareness of Holistic Contagion model and the accompanying scale. Next, we briefly define job burnout, job satisfaction and organizational commitment and delineate arguments to formulate hypotheses regarding the effects of the new contagion leadership’s dimensions on followers’ well-being and work attitudes, and assess the nomological/criterion validity of the new scale. Finally, we present two validation studies of the new Leader Awareness of Holistic Contagion Scale using a multi-sample and cross-country research design, and describe the scale development and validation process (e.g., Cortina et al., 2020; Hinkin, 1998).

## 2. Emotions and Leadership

Despite the existence of different approaches to leadership, a converging view of the literature tends to see the leader as someone who uses their influence to channel followers’ efforts to achieve organizational goals, as proposed by Yukl and van Fleet (1992). Consistently, leadership is considered a process, and leaders are key agents who have technical (e.g., strategizing) and social (e.g., influencing) responsibilities to ensure followers function effectively (Jex & Britt, 2008).

Research on leadership effectiveness has increasingly brought to the forefront the leader’s use of emotions as a requirement for overcoming difficulties and efficient functioning that underpin good performance (Halimah et al., 2014). The leadership literature has extensively acknowledged the key role of emotions and how leaders may use emotions to enhance effective leadership and achieve success (e.g., Antonopoulou, 2024; Halimah et al., 2014). Below, we briefly review different leadership models concerned with relational and emotional aspects, and related operationalization, and present the theoretical foundations that underpin the development of our overarching conceptualization and measurement of leadership grounded in emotional contagion dynamics of emotion exchanges among multiple stakeholders in the workplace.

### 2.1. Overview of Leadership Models and Emotion Management

This section provides an overview of common leadership models among traditional and emerging approaches (i.e., leader group prototypicality, leader–member exchange, authentic, transactional, transformational, and servant leadership) in the existing literature (e.g., Dinh et al., 2014; van Knippenberg & Hogg, 2003) and comments on the extent to which they involve emotion-related aspects. Moreover, the construct of group member prototypicality (Hogg, 2001) will be outlined as a salient relational construct that is explicitly differentiated from leadership processes and thus will be considered for the purpose of discriminant validity.

*Leader Group Prototypicality* (LGP; e.g., Hogg, 2001) is a social identity approach to leadership and, therefore, intrinsically concerned with social and relational aspects. Specifically, this theoretical framework aims at defining the leader based on their key features and the extent to which they are representative of the group they lead. To fully understand leadership processes, it is critical to consider the part played by the leader and followers’ common group membership (i.e., LGP) and to acknowledge that leadership processes are carried out within the framework of this shared social identity (e.g., van Knippenberg & Hogg, 2003). Thus, this leadership model informs us of the similarities between the leader and their group and postulates it as an implicit factor of leaders’ effective action grounded in social bonding and cohesion as the glue of leader–follower synergy. While LGP does not capture a specific leadership style, it does signal the extent to which the leader is representative of a collective identity and, therefore, is trusted more to act in the group’s best interest (Cicero et al., 2010). Yet, LGP does not explicitly refer to emotions within the leadership latitude and does not operationalize this aspect.

On a different, yet related note, the construct of *group member prototypicality* (GMP) describes a person’s identification with emerging social group prototypical traits that dictate members’ conduct, emotions, and ideas in a particular setting (Tajfel & Turner, 1979). Beliefs, emotions and behavior are more influenced by group membership the more people identify with the group and define themselves in terms of the group identity (i.e., group member prototypicality; Hogg, 2001). As such, the stronger the social identity, the more uniform and homogenous the behaviors of all group members (Kaiser et al., 2008).

Overall, while LGP focuses on how the leader is perceived as similar to their group and identifies with it, GMP focuses on how team members perceive themselves as similar to their group and identify with it. In this paper, we considered GMP because the social identity literature qualifies it as a workgroup/relational construct while also explicitly differentiating it from leadership processes (Cicero et al., 2010; Steffens et al., 2021). Thus, GMP will be considered for discriminant validity purposes of leadership models and as a non-leadership construct.

Similar to LGP, the *leader–member exchange* model (LMX; Graen & Uhl-Bien, 1995) is concerned with social processes and, specifically, the leader–follower relationship. According to the LMX model, the leader is the one who offers collaborators liberty, responsibility and support in return for their dedication. In order to provide ingroup members additional responsibilities, autonomy in their tasks, rewards, and attention, the leader builds a differential relationship of more trust with select collaborators (i.e., ingroup) than with others (i.e., outgroup). The four primary dimensions or types of relational exchanges that are key in the LMX model are (Dienesch & Liden, 1986; Pellegrini et al., 2010; Sin et al., 2009): contribution (i.e., leader–follower joint activities), loyalty (i.e., leader–follower mutual support), affection (i.e., leader–follower mutual satisfaction) and professional respect (i.e., leader–follower mutual appreciation of professional expertise). While the LMX approach includes affect-related aspects such as empathic concern for followers and consistently operationalizes them, it does not explicitly theorize emotion management nor tap into emotions in its measurement.

According to Gardner et al. (2005), *authentic leadership* is characterized by a leader who builds open and honest relationships with his or her own team members, encourages self-improvement and a positive environment, and has a positive moral outlook with high ethical standards that direct decision-making. Five distinct components shape the leader’s action in the authentic leadership model developed by Walumbwa et al. (2008): internalized regulation (i.e., the capacity to self-regulate), self-awareness (i.e., the capacity to comprehend and interpret the environment and recognize the impact it has on others), relational transparency (i.e., the capacity to be genuine and open, fostering trust in collaborators), balanced information processing (i.e., the capacity of the leader to impartially evaluate all pertinent factors prior to making a decision) and positive moral perspective (i.e., the capacity to have a positive vision based on ethics and integrity). Although the measurement of authentic leadership includes the leader’s management of relational aspects, it does not explicitly theorize emotion management nor tap into emotions in its measurement.

Among contingent approaches emphasizing that the leader needs to adapt to various situations, the *transactional leadership* model (Bass & Avolio, 1994) is concerned with rewarding or disciplining employees based on their work output. Transactional leaders place a high value on job standards, task accomplishment, and employee compliance. The two principles of this leadership model are contingent reward (i.e., recognition of the followers’ efforts and good performance) and management-by-exception (i.e., the leader’s intervention only when corrective action is needed). As such, it does not involve relational or emotional aspects in its theorizing and measurement. Differently, the *transformational leadership* model (Bass & Avolio, 1994) was developed to capture a leader’s charisma in creating excitement among followers, promoting fresh perspectives on the job, letting employees know the company’s goals, assisting in the development of employees’ capabilities, and inspiring team members. Some key components of the transformational leadership style are (Podsakoff et al., 1990): (1) identifying and articulating a vision or rather, inspiring others with their dreams; (2) providing an appropriate model; (3) fostering acceptance of group goals, which refers to the leader’s behaviors that tend to encourage cooperation among employees; (4) high performance expectations; (5) intellectual stimulation or rather, encouraging staff members to reconsider their beliefs in order to approach long-standing issues at work in a fresh way; and (6) offering individualized support, which refers to the leader’s actions that show respect for his staff members and a genuine interest in their needs and feelings. As such, transformational leadership marginally includes personal thoughtfulness and the use of emotional skills (Furtner et al., 2010) and explicitly refers, in its theorization and measurement, to leaders’ consideration and management of followers’ emotion-related aspects.

Finally, the *servant leadership* model (Greenleaf, 1998) is centered on followers’ well-being and concerned with both relational and emotional aspects in lead-follower interactions. A leader who practices servant leadership shares authority and prioritizes the needs of others over their own, assisting collaborators in developing and expressing their potential as well as in elaborating emotional experiences. The overarching theoretical framework developed by Barbuto and Wheeler (2006) encompasses the following eleven dimensions: (1) calling (i.e., the desire to serve and the willingness to sacrifice one’s interests for the sake of others); (2) listening (i.e., the capacity to listen to the ideas and suggestions of others); (3) persuasion; (4) conceptualization (i.e., the capacity to encourage followers to use creative processes); (5) foresight (i.e., the capacity to outlook future scenarios; (6) community building (i.e., the capacity to develop an organizational spirit); (7) stewardship (i.e., the capacity to read the stratified needs of the organization and society at large); (8) growth (i.e., helping followers to grow in a positive direction); (9) awareness (i.e., the capacity to observe what is happening by collecting cues from the environment); (10) empathy (i.e., the capacity to appreciate the circumstances of others); and (11) emotional healing (i.e., the capacity to understand how and when to find a solution (or emotional healing) to employees experiencing emotional hardship). Empathy and emotional healing, in particular, are the most novel and distinctive leadership aspects suggested by this model. As such, servant leadership qualifies as a leadership model that involves emotion management and coherently operationalizes this aspect.

Overall, we can draw the following conclusions. First, the existing leadership models and related scales do not capture the leader’s management of emotions, except the servant leadership style explicitly embedding the emotional healing skills of the leaders (e.g., Barbuto & Wheeler, 2006), and the transformational model that is partly concerned with followers’ feelings (e.g., Podsakoff et al., 1990). Second, both servant and transformational leadership include leaders’ emotion management by focusing exclusively on the followers’ affect-related issues, but do not involve the leader’s awareness and/or management of their own emotions. Servant leadership explicitly refers to leaders’ awareness, but only in terms of general situational awareness.

### 2.2. Integrating Emotional Contagion into Leadership Theorizing

A major emotion-related factor pervasively studied in association with leadership is the leader’s intelligent use of emotions, or rather, emotional intelligence (EI; Goleman, 1995), defined as the ability to understand and manage one’s own emotions as well as the emotions of others. According to Goleman (1995), emotional intelligence builds on five fundamental components: *self-awareness* (i.e., the ability to recognize and understand one’s own feelings, strengths, weaknesses, values, and goals), *self-regulation* (i.e., the ability to effectively control one’s emotions, urges, and behaviors while exhibiting flexibility in response to changing external circumstances), *motivation* (i.e., the internal drive that encourages people to pursue goals and pursue personal and professional growth despite challenges or disappointments), *empathy* (i.e., the cognitive and affective ability to understand and relate to people’s feelings, needs, and perspectives and then respond appropriately to their emotional states), and *social skills* (i.e., effective communication, building and maintaining relationships with others, and working cooperatively with others). Sundry studies and meta-analytic findings (e.g., Augusty & Jain, 2019) have shown that EI may impact leadership skills and action by facilitating conflict management and the handling of stressful situations that underpin the task execution of followers. As such, EI is a leader’s personal attribute and a factor external to leadership in itself.

While the current study does not focus on EI, it is crucial to critically review its role within the overarching topic of the link between emotions and leadership. In this regard, three issues are of paramount relevance.

First, EI stands as a major factor in conjunction with leadership and is a separate construct not intrinsically embedded in leadership models and their measurement. Second, while EI variously refers to the skills and/or dispositions of the leader (e.g., Lubbadeh, 2020), it is nonetheless a within-individual factor that focuses on leaders’ sensitivity towards affect-related phenomena and frames emotions as static events alternatively experienced by followers and/or the leader. Third, differently from EI, emotional contagion (i.e., the involuntary absorption of other people’s emotions during social encounters; Hatfield et al., 1993) shifts the focus of emotional processes from inside the leader or the followers (i.e., an *intra-individual* perspective) towards contextual dynamics of emotional exchanges (i.e., an *inter-personal* perspective) occurring between leader and followers, but also occurring with other factual stakeholders of the workplace such as clients (or externals to the organizations with whom members interact).

Table 1 summarizes a conceptual comparison between the three key emotion-related constructs of emotional contagion, empathy, and emotional intelligence, frequently yet variedly studied in conjunction with leadership. Overall, while contagion refers to the *transfer* of emotions and occurs involuntarily, both empathy and EI are voluntary processes (also underpinned by different neural mechanisms in comparison to contagion), respectively involving the *understanding* of others’ emotions and the *use* of one’s/others’ emotional states. Interestingly, the pre-reflective nature of emotional contagion qualifies it as a precursor of empathy, which, in turn, is pointed out by the literature (e.g., Goleman, 1995) as a sub-dimension/facet of EI.

The present paper engages an inter-personal approach to the study of emotions and leadership, and switches from dispositional approaches to emotions (e.g., emotional intelligence) towards contextual emotion-related factors as proposed by emotional contagion dynamics. We briefly review the state of the art in the research of emotional contagion at work and then provide an overarching framework of leadership processes encompassing emotional contagion management that builds upon the existing leadership and contagion studies.

Emotional contagion refers to how people absorb the emotions experienced by others around them and, therefore, encompasses the inter-personal aspect of the emotions shared during social interactions (Hatfield et al., 1993). The literature suggests that people involuntarily detect emotional clues associated with the emotional states of others expressed through facial, verbal, and postural inputs, and automatically imitate such behavioral, vocal, facial, and postural emotional cues, thus synchronizing and attuning with their feelings and involuntarily “catching” their emotions (Hatfield et al., 1994). Moreover, EC can also spread through textual and visual cues in electronic communication contexts in addition to face-to-face situations (Belkin, 2008). According to research on the neurological underpinnings of EC, it spreads milliseconds before conscious awareness (LeDoux, 2002). Specifically, it takes a few milliseconds for the emotional signal to enter the neocortex for awareness (LeDoux, 2002), enabling a person to be conscious of their emotional interactions with other people. As a result, people in the workplace can become aware and report on their experiences with contagion of emotions absorbed by different stakeholder sources in their context (e.g., colleagues, clients, leaders).

Research on emotional contagion at work has variously and selectively focused on the study of emotions exchanged between employees and their clients in productive settings (e.g., Pugh, 2001), among employees themselves as is the case of work teams (e.g., Barsade, 2002), and between leaders and followers by engaging both the perspective of downward contagion of emotions from the leader to their followers (e.g., Bono & Ilies, 2006) or upward contagion of the emotions that the followers infect in their leader (e.g., Dasborough et al., 2009). Along with a heterogeneous and parceled research inquiry of EC occurring within dyadic relationships such as employee–client, employee–employee, and leader–follower interactions, other research has engaged a holistic perspective (e.g., Bono & Ilies, 2006; Inness et al., 2008; Petitta & Naughton, 2015) and examined the simultaneous spreading of emotional exchanges among all of these different workplace stakeholders thus mapping emotional processes or EC within a multi-foci framework that accounts for contextual emotional dynamics or rather, the different stakeholders sources of emotions and the multiple relational pathways through which emotions spread in the whole organizational setting. As noted, “holistic” contagion emphasizes the study of emotional exchanges beyond the more common two-person interactions. That is, rather than surveying organizational members selectively and exclusively on the extent to which they feel inundated by a client’s emotional state or their leader’s mood, the holistic investigation of contagion simultaneously probes whether one’s emotional state is absorbed by a client or by the leader or by other colleagues and calls the respondent’s attention on all possible sources (i.e., stakeholders) of absorbed emotions in their work environment.

Within the realm of holistic emotional contagion, a study on 694 workers from different organizations examined both positive (i.e., joy) and negative (i.e., sadness, fear, anger) emotions that employees reported to absorb from others at work, as well as whether these emotions were absorbed by one’s colleagues and/or leaders and/or clients with whom employees interacted daily (Petitta & Naughton, 2015). Recall that emotional contagion is an involuntary absorption of others’ emotions (not a voluntary use of emotion as in emotional intelligence or empathy) and that holistic mapping allows employees to indicate the sources of the “second-hand” emotions (Petitta et al., 2021) that they experience as a consequence of social exposure to others at work. Results have shown that across different organizations and industry sectors, the majority of the emotions taken into account for contagion were more commonly absorbed by coworkers and clients, whereas, counterintuitively, leaders were the second to the least associated with emotional exchanges.

The result that leaders rank second to third as the source of contagion during emotional exchanges after coworkers of their followers (i.e., the followers among themselves) and, sometimes even after external stakeholders such as clients (with whom leaders and followers both interact) has the following implications for leadership: (a) followers experience higher emotional intimacy among themselves and more emotional distance from their leader, (b) although the leader has a marginal position within the “emotional orbit” of their followers, “emotional orbit” and “power/influence orbit” are two different (yet, interrelated) domains of leadership processes, (c) the leader–follower(s) relationship does not occur in a vacuum and both parties interact with other parties (e.g., clients), thus structuring emotional exchanges according to patterns other than the traditional influence or power criteria within the leader–follower “bubble”, and (d) given the well-established relevance of emotions for leadership action, the leader’s marginal role should not be undervalued and leaders should be made aware of their marginal position due to the systematic emotional primacy of coworkers; more generally, leaders should be mindful of their emotional positioning within the extended field of emotional exchanges among all stakeholders who are relevant for successful performance.

In the following section, we delve into each point and build upon the leadership and contagion studies to propose a conceptual framework that embeds leaders’ mastery of contagion dynamics and that complements existing leadership models by incorporating contextual emotions’ management dimensions.

### 2.3. Measuring Leaders’ Management of Emotions Within an Emotional Contagion Framework: The Leader Awareness of Holistic Contagion Scale

The Leader Awareness of Holistic Contagion model (i.e., LAHC) is theoretically grounded in the leadership and emotional contagion studies. The LAHC is a holistic and multi-foci (i.e., different sources of absorbed emotions) emotional leadership model that refers to leaders’ awareness of and willingness to manage holistic emotional exchanges among different roles (i.e., multiple stakeholders such as leaders, colleagues and clients) in their own context. The LAHC conceptualization may complement existing leadership models by (a) shifting the focus of leadership emotional processes towards an inter-personal perspective of emotion exchanges among people in the workplace (as opposed to within-person emotional processes such as emotional intelligence), (b) contextualizing emotional exchanges within a broader “emotional orbit” including multiple stakeholders at work (e.g., leaders, followers, clients) beyond the leader–follower(s) dyad, (c) assessing leaders’ awareness of the emotional traffic among multiple stakeholder and their positioning within the emotional orbit of their followers (i.e., leader–follower(s) emotional closeness/distance), and (d) capturing leaders’ active exploration of contextual emotions exchanged among multiple stakeholders as well as their willingness/intention to include the management of contagion dynamics in their leadership practice.

The LAHC model is a four-dimensional leadership construct, with each dimension capturing a specific facet of a leader’s mastery of contagion dynamics in the workplace, derived from the above literature review and research findings.

Specifically, *leader awareness of holistic emotional exchanges* captures the extent to which a leader is aware of holistic emotional exchanges among different roles in their own context beyond the leader–follower(s) dyad (i.e., not just between him or her and their follower(s)). Notably, a leader’s awareness of emotional traffic is in line with the self-awareness concept, a key factor proposed by the emotional intelligence literature (i.e., the leader’s capacity to acknowledge and comprehend their emotions; Goleman, 1995); yet, while the latter provides a within-leader individual-centric perspective, the LAHC’s awareness is inter-personal and context-focused.

Next, the *exploration of holistic emotional exchanges* captures the extent to which a leader actively explores and talks with their followers: (a) about the reasons why they feel much closer and more in confidence witheach other, rather than in them; and (b) on how comfortable the followers feel with them and sharing their emotions, as compared to what they do with colleagues or clients. Similar to the social skills proposed by emotional intelligence for effective leadership (Goleman, 1995), a leader’s probing of emotional exchanges around them contributes to building contextual awareness and relationships grounded in genuine interest for emotional ambience that supports task performance.

Moving further, the *leader–follower(s) emotional distance awareness* conceptualizes the extent to which a leader is aware that emotional exchanges and familiarity are higher among followers themselves rather than between the leader and follower(s). As noted, emotional magnets assessed by contagion research (e.g., Bono & Ilies, 2006; Petitta & Naughton, 2015) assign to the leader a secondary or marginal role in the affective life of employees at work. However, the emotional marginality of a leader is not to be equated with their power marginality. Specifically, power is frequently and variously defined as the tendency to *influence* the behavior of others with or without resistance (Fisk & Friesen, 2012). Moreover, social influence takes many forms and can be seen in conformity, obedience and leadership, and occurs when one’s *emotions*, opinions, or behaviors are affected by others (Kelman, 1958). Thus, emotions and power are different constructs; yet, emotions are partially embedded in the concept of power/influence. Overall, the leader’s awareness of followers’ emotional distance may contribute to differentiating “emotional orbit” from “influential orbit” and to examining how they intersect and diversely contribute to the leader’s action.

Finally, *willingness to include “LAHC”* captures the extent to which a leader is willing and able to adjust their behavior by including active explorations of holistic emotional exchanges among different roles in their own context. Specifically, while contagion dynamics are seldom considered by leadership models, a leader’s orientation to behavioral change beyond common leadership practices may inform leadership action according to patterns other than the traditional influence or power criteria.

Consistent with Podsakoff et al.’s (2016) recommendations, we specify the type of property of the new construct (i.e., the nature of the focal concept), as well as its entity (i.e., the object or event to which the property applies), attributes (i.e., the essential and unique characteristics), dimensionality, and differences from other similar concepts. In terms of the *type of property*, the leader’s awareness of holistic contagion (i.e., focal concept) refers to both the person’s awareness (i.e., the mental state of being cognizant of perceived external events and internal stimuli; American Psychological Association, 2018) and willingness/intention (i.e., a mental state in which a person commits to a course of action and distressing tasks; Koole et al., 2023; Reid et al., 2017). Therefore, the *entity* to which the leader’s awareness of holistic contagion applies is the person’s (i.e., the leader) characteristics in terms of their awareness and willingness/intentions. The specific *attributes* of the construct are: (a) awareness of emotional exchanges among people at work (i.e., contagion of emotions not simply emotions in oneself or others); (b) situation awareness including multiple stakeholders, such as clients, beyond the dyadic leader–follower relationship; and (c) openness (i.e., willingness/intention) to include the management of emotional exchanges in one’s leadership practice.

Table 2 summarizes how the LAHC model structurally and functionally diverges from common relational/emotional leadership models, such as LMX, servant, and transformational leadership. Specifically, the leader awareness of holistic contagion is a multi-faceted construct (*dimensionality*) and differs (*differences from similar constructs*) from other leadership models and/or emotion-related constructs applied to leadership (e.g., leader’s emotional intelligence) because it is not simply emotional awareness (i.e., the ability to recognize and understand emotions in oneself and others) as in emotional intelligence or empathy (i.e., within-person perspective) but it is the perception of contagion of emotions or rather, emotions that people absorb from others and exchange each other during social intercourses (i.e., inter-personal perspective), thus collectively converging on the same emotions and sharing the same feelings during social interactions at work. As such, it refers to the awareness of the composite “emotional traffic” that occurs in the workplace, and not simply one’s or others’ emotions. As shown in Table 2, it is focused not simply on exchanges between leader and followers (only two types of stakeholders) but among multiple stakeholders (i.e., multi-foci and holistic contagion) that populate the social context at work (e.g., clients). Finally, the leader’s awareness of holistic contagion is also different from other leadership models, as the construct is fully focused on examining the leader’s mastery of contagion dynamics at work and their openness to commit to managing such contagion dynamics (i.e., a leadership model fully focused on and embedding emotion management). Conversely, LMX tangentially mentions emotional processes by considering the leader’s affection (i.e., mutual leader–member satisfaction), whereas transformational leadership only focuses on motivational processes. Servant leadership is the only model partially including emotional process in its conceptualization by considering leader’s emotional support of their followers; yet, emotional mechanisms are explored only within the leader–follower(s) dyad.

Overall, the hallmark of the LAHC model is the potential to call the leader’s (and followers’) attention to all possible sources (i.e., stakeholders) of emotions exchanged in their work environment, beyond the short-sided dyadic relationship with their followers.

The development of the Leader Awareness of Holistic Contagion Scale (LAHCS) is theoretically grounded in the LAHC conceptualization and the leadership and emotional contagion aspects derived from the above review of the literature (i.e., deductive approach; Hinkin, 1998). We developed the scale to capture the key conceptual features of the new LAHC leadership model. Consistent with the LAHC conceptualization, the LAHCS taps into the following four dimensions that cover a leader’s mastery of holistic emotional exchanges simultaneously occurring among multiple stakeholders: (1) leader awareness of holistic emotional exchanges, (2) exploration of holistic emotional exchanges, (3) leader–follower(s) emotional distance awareness, and (4) willingness to include “LAHC”. The details of the scale development are provided in the Method section of Study 1, as a first step of the whole validation process (e.g., Cortina et al., 2020; Hinkin, 1998).

### 2.4. The Relevance of Emotions for Leadership and Its Outcomes

An accumulating body of evidence and meta-analytic findings (e.g., George, 2000; Augusty & Jain, 2019) suggest that leaders with competences encompassing the ability to understand and manage their own and others’ emotions are better able to deal with conflicts and setbacks, remain in control of their demeanor when negative feelings arise, encourage people when they are down, and build positive and productive connections, thus achieving a more effective leading action and creating a positive environment that reduces stress levels among followers and increases their well-being (Goleman et al., 2002; York, 2022).

According to Maslach (2003), job burnout is a stress-related syndrome caused by extended exposure to work-related stressors. Emotional exhaustion (i.e., a state of physical and emotional depletion due to regular and overwhelming demands that make employees feel left with little or no energy to devote to their jobs) and cynicism (i.e., a psychological detachment and a negative attitude toward one’s work and workplace due to a defensive distance from excessive emotional demands at work), are the two main manifestations of burnout (Maslach & Leiter, 2008).

Indeed, leaders who are better equipped to accurately perceive and manage emotions build genuine connections and create an environment of support that helps employees thrive at work and prevents them from experiencing emotional weariness and disinterest in work (i.e., burnout; e.g., McGrath, 2025; Thomas, 2018). Moreover, sundry studies have demonstrated that a leader’s emotions and sentiments are contagious, and when the leader is feeling “upbeat”, followers will capture that feeling, have greater self-assurance in their performance abilities and responsiveness toward organizational goals (Goleman et al., 2002). Given the above literature, we argue that leaders who are mindful of the emotional traffic among multiple stakeholders have a more expanded perception (i.e., inter-personal perspective) and understanding of emotions that impact team dynamics and are able to create a positive and healthy work environment that poses fewer emotional demands and prevents followers from experiencing burnout. Thus, we hypothesize the following.

**Hypothesis** **1.** *The four LAHCS dimensions will be negatively associated with (a) emotional exhaustion and (b) cynicism.*

As noted, a leader’s skills in managing emotions play an integral role in the workplace because the ability to gauge oneself and one’s followers emotionally helps utilize emotions as a guiding tool for inter-personal effectiveness within the social environment (Kunnanatt, 2004) and fosters the necessary social skills to succeed in a professional context (Dong & Howard, 2006). Specifically, leaders with high emotion management competences possess the skills to sense how their followers feel about their work situation and to intervene effectively when they feel dissatisfied (Mayer et al., 2000), thus producing positive work attitudes and job satisfaction (i.e., a pleasurable emotional state resulting from the appraisal of one’s job experiences; Locke, 1976) among employees (Carmeli, 2003; Sy et al., 2006; Wong & Law, 2002). Similarly, the literature (Gardner & Avolio, 1998) suggests that a leader’s self-monitoring ability to regulate their behavior within the leader–follower relationship may enhance followers’ commitment (i.e., employees’ attachment to and identification with the organization; Allen & Meyer, 1990). Moreover, a leader’s effective management of emotional processes builds a positive environment that helps employees feel involved and experience higher commitment towards the organization (e.g., Sy et al., 2006). Consistently, we argue that a leader’s mastery of complex contagion processes within the social context, including multiple stakeholders, further strengthens their ability to regulate their behavior and improve communication with followers and social networking, thus facilitating employees’ sense of involvement with the task at hand, and attachment to the organization and organizational goals. Consistent with the above arguments, we expect to find the following.

**Hypothesis** **2.** *The four LAHCS dimensions will be positively associated with followers’ (a) satisfaction with their leader and (b) affective organizational commitment.*

## 3. Study 1: Construct, Convergent, and Discriminant Validity

Study 1 was conducted to provide evidence for the construct, convergent, and discriminant validity of the LAHCS following the steps recommended by Hinkin (1998), including item generation (Step 1), questionnaire administration (Step 2), initial item reduction (Step 3), confirmatory factor analysis (Step 4), and convergent and discriminant validity (Step 5). Specifically, we explored the factorial structure of the LACHS to examine construct validity. Next, we examined the correlations between the LAHCS scores and the transformational, transactional, servant, authentic leadership, LMX, and leader group prototypicality scales to assess convergent validity. Moreover, we tested discriminant validity by running the additional correlations between the LAHCS dimensions and group member prototypicality, which measures how team members perceive themselves as similar to their group and identify with it. From a theoretical standpoint, given that leadership is considered a social role (Kerr et al., 2006), group member prototypicality qualifies as a relational construct while also capturing a social mechanism different from the leadership process (Steffens et al., 2021).

### 3.1. Method

#### 3.1.1. Sample and Procedure

The sample included 1454 employees from various Italian organizations. The survey data were anonymous and collected cross-sectionally at a single point in time from a sample of Italian adult workers. The sampling technique was based on a convenience sample strategy. The research team contacted potential participants belonging to the same organization to request their participation in the study based on the opportunity and accessibility of the study’s participants. The research team approached administrators within each organization. Upon reaching agreement on participation, the research team provided information sessions to describe the project, encourage participation, and address concerns from potential participants. Participation was voluntary, anonymous, and not rewarded with any incentive. The study followed the guidelines of research ethics in compliance with the Ethical Principles of the Helsinki Declaration of 1964, in order to protect individual participants from any form of potential physical and/or emotional harm. Participants were provided with informed consent that explained the anonymous nature of the data collection and their rights as research participants. The research team distributed questionnaires at each location, and most participants completed and returned the survey (in a sealed envelope) on the same day.

Participants’ ages ranged from 19 to 71 years, with a mean age of 37.68 years (*SD* = 11.57). With regard to gender, 65.3% were male, 33.6% were female, and 1.0% chose not to answer this question. With regard to education, 66.2% completed high school, 16.9% college, 10.3% junior high school, 3.3% specialization, and 3.4% left this question blank. The mean tenure was 11.73 years (*SD* = 11.27). Fifty-three percent of the sample worked for a private company while 46.2% worked for a public organization. Most participants (57.8%) held a non-managerial position while 30.9% left this question blank. Regarding type of contract, 39.2% of the sample had a permanent position while 23.9% had a temporary position and 36.9% chose not to answer this question. While the questionnaire did not include items on the organization type, the respondents that accepted to participate to the study belonged to organizations in the occupational areas of transportations, retail, finance, communication, health services, public administration, and the military.

#### 3.1.2. Transparency and Openness

The covariance matrix and analysis code are available upon request from the first Author. Row data for this study are not available as we do not have permission from participants for row data sharing.

### 3.2. Measures

Below is a description of the newly developed Leader Awareness of Holistic Contagion Scale and the measures used in the data collection for the current analyses.

**Leader Awareness of Holistic Contagion Scale.** The development process of the Leader Awareness of Holistic Contagion Scale (LAHCS) followed the steps recommended by Hinkin (1998).

*Step 1: Item Generation.* As recommended (Hinkin, 1998, p. 2), we first sought to develop items that “will result in measures that sample the theoretical domain of interest”, thus adequately representing the construct under investigation. Consistently, using a “deductive approach” (Hinkin, 1998, p. 3) to generate the initial set of items, the LAHCS items’ development was theoretically grounded in the above literature review and related constructs defined by the LAHC multifaceted model. Specifically, the scale was developed to capture a leader’s awareness of how contagion of emotions is pervasive and spreads among multiple stakeholders within the workplace, such as followers and clients. Moreover, the scale captures the leader’s mastery of contagion dynamics given their relevance in shaping the leader’s “grip” on their followers and influential latitude that underpins effective leader actions. Finally, consistent with the multifaceted structure of the LAHC model presented above, the scale included four subdimensions (i.e., leader awareness of holistic emotional exchanges, leader–follower(s) emotional distance awareness, exploration of holistic emotional exchanges, and willingness to include “LAHC”).

Overall, the LACHS consists of 14 items across four subscales, each measuring a facet (i.e., focal variables) of a leader’s mastery of emotional contagion dynamics: (a) three items of the Leader Awareness of Holistic Emotional Exchanges subdimension aim at capturing the extent to which the leader is aware of multiple emotional exchanges such as emotions mutually exchanged between him or her and their followers and/or clients and/or supervisors (a sample item is “*My leader is aware of the emotions that we mutually infect each other with*”); (b) six items of the Leader Exploration of Holistic Emotional Exchanges subdimension measure the extent to which the leader actively probes with their followers whether the emotions that they feel at work are more associated with other stakeholders (e.g., teammates, clients, other superiors) rather than the leader themselves and whether there is greater confidence and relational closeness among them rather than with him/her (a sample item is “*My leader explores with me if I happen to experience more emotional contagion with my clients, rather than with her/him*”); (c) three items of the Leader Awareness of Emotional Distance subdimension measure the extent to which the leader is aware of the greater intimacy that the followers experience among themselves rather than with him/her (a sample item is “*My leader is aware that I feel emotionally more distant from her/him in comparison to my colleagues*”); and (d) two items of the Leader Orientation to Include “LAHC” subdimension aims at examining whether the leader would be willing and able to manage the holistic contagion dynamics that occur within the workplace, even when he or she is not fully aware of them (a sample item is “*Even if my leader were not aware of the emotional exchanges that occur within our work context, he or she would be able to include the management of this aspect in their leadership behaviors*.” While a minimum of three items per scale is commonly recommended (e.g., Marsh et al., 1998), there are no hard-and-fast rules guiding this decision (Hinkin, 1998). More importantly, consistent with the recommended principle of parsimony in scale development (e.g., Thurstone, 1947), a typical milestone in item creation (Hinkin, 1998) is to ensure that the domain under study is adequately sampled and that the items reasonably represent the construct under examination. Indeed, two-item scales are valid and acceptable measures as long as the semantic representations of *items* connect to *scale* characteristics such as Cronbach’s alpha internal consistency (e.g., Eisinga et al., 2013).

Items were all positively worded and randomized to avoid response set (Cronbach, 1946). Respondents were asked to rate the statements on a 5-point frequency scale ranging from 1 (*Never*) to 5 (*Always*).

**Transformational Leadership.** Transformational leadership was measured using 23 items from the Transformational Leader Behaviors Scale (Podsakoff et al., 1990), assessing the leader’s charisma, leading by providing a model and individualized support, and fostering group goal acceptance. Responses were made on a five-point frequency scale from 1 (*Never*) to 5 (*Always*). A sample item was “*My leader provides a good model for me to follow*.”

**Transactional Leadership.** Transactional leadership was measured using five items from the Transactional Leader Behaviors Scale (Podsakoff et al., 1990) assessing leaders’ contingent rewards and management-by-exception behaviors. Responses were made on a five-point frequency scale from 1 (*Never*) to 5 (*Always*). A sample item was “*My leader always gives me positive feedback when I perform well*.”

**Leader–Member Exchange.** Leader–member exchange between respondents and their leaders was measured using seven items from the leader–member exchange scale (Graen & Uhl-Bien, 1995) assessing leaders’ respect, trust and obligation. Responses were made on a five-point frequency scale from 1 (*Never*) to 5 (*Always*). A sample item was “*My leader understands my job problems and needs well*.”

**Servant Leadership.** Servant leadership was measured using 13 items from the altruistic calling, emotional healing and wisdom dimensions of the Servant Leadership Scale (Barbuto & Wheeler, 2006). Responses were made on a five-point frequency scale from 1 (*Never*) to 5 (*Always*). A sample item was “*My leader is good at helping me with my emotional issues*.”

**Authentic Leadership.** Authentic leadership was measured using eight items from the Authentic Leadership Questionnaire (Walumbwa et al., 2008) assessing leaders’ self-awareness, relational transparency, internalized regulation (i.e., authentic behavior) and balanced processing of information. Responses were made on a five-point frequency scale from 1 (*Never*) to 5 (*Always*). A sample item was “*My leader seeks feedback to improve interactions with others*.”

**Leader Group Prototypicality.** Leader group prototypicality was assessed using four items of the Italian version of the scale from Cicero et al. (2007) that capture followers’ perception of the extent to which their leader holds prototypical characteristics of their group and is representative of the group identity shared among teammates. A sample item was “*My team leader is a good example of the kind of people that are member of my team*.” Responses were rated on a six-point scale ranging from 1 (*strongly disagree*) to 6 (*strongly agree*).

**Group Member Prototypicality.** Group member prototypicality was assessed using five items of the Italian version of the Team Identification scale from Cicero et al. (2007) and measures the extent to which team members identify with prototypical characteristics of their group. A sample item was “*When I talk about my work team, I usually say “we” rather than “they””.* Responses were rated on a six-point scale ranging from 1 (*strongly disagree*) to 6 (*strongly agree*).

### 3.3. Analytical Strategy

In order to cross-validate findings obtained from one sample with an independent sample, we randomly split the whole sample into two approximately equal groups. We conducted an explanatory factor analysis (EFA) on the first subsample (*n* = 753) using M*plus* 7 (Muthén & Muthén, 1998/2015) to explore the number of factors underlying the 14 LAHCS items. We then cross-validated (Stone, 1974) the resulting factor structure with the second subsample (*n* = 701) using confirmatory factor analysis (CFA). The following graded criteria were used to evaluate the quality of each examined model: the root-mean-square error of approximation (RMSEA) ≤ 0.08, the standardized root-mean-square residual (SRMR) ≤ 0.10, and the Bentler Comparative Fit Index (CFI) ≥ 0.90 for acceptable fit; RMSEA ≤ 0.05, SRMR ≤ 0.08, and CFI ≥ 0.95 for good fit (Browne & Cudeck, 1993; Hu & Bentler, 1999). The M*plus* robust maximum likelihood estimation procedure (i.e., MLR estimation) was used for the analyses.

Before examining the discriminant and convergent validity of the LAHCS, we conducted a CFA to examine the construct validity of leadership scales (i.e., transformational leadership, transactional leadership, leader group prototypicality, leader–member exchange, authentic leadership, and servant leadership) and group member prototypicality (i.e., non-leadership construct) using the complete Italian sample (*N* = 1454). Following that, with the complete Italian sample (*N* = 1454), we examined the discriminant validity of the LAHCS from group member prototypicality using CFA. Convergent validity was demonstrated by high (disattenuated) correlation coefficients for the relationships between four LAHCS dimensions and other leadership scales (Nyklíček & Denollet, 2009), whereas discriminant validity was supported by low (disattenuated) correlation coefficients for the relationship between four LAHCS dimensions and GMP. To control for error methods, we corrected zero-order correlations for the reliability using the formula provided by Murphy and Davidshofer (1988). Specifically, the disattenuated correlation is the raw correlation between x and y divided by the square root of the product of the reliability of x and the reliability of y.

### 3.4. Results

*Step 3: Initial Item Reduction*. To test the dimensionality of the LAHCS, all 14 items were submitted to a preliminary EFA on the total sample (*N_total_* = 1454). Initially, four factors were extracted, which together accounted for 71.37% of the variability. The solution showed all eigenvalues >1. Factor 1, designated exploration, was composed of items 4, 5, 9, 10, 11, and 2, and accounts for 39.72% of the variability. All loadings were significant and above 0.30 (Tabachnick & Fidell, 1996), and were retained as the factor was defined solely by appropriate marker items. Factor 2, designated exchanges awareness, was composed of items 7, 8, and 6, and accounts for 14.2% of the variability. All loadings were significant and above 0.30, and were retained as the factor was defined solely by appropriate marker items. Factor 3, designated prevention, was composed of items 14, 13 and 12, and accounts for 10.29% of the variability. All loadings were significant and above 0.30. In order to have the factor defined solely by appropriate marker items (e.g., Hinkin, 1998) and increase the LAHCS content validity, item 12 (“My leader is aware that her/his power to influence us as followers does not automatically correspond to an emotional influence on us”), originally developed for the “distance awareness” sub-dimension, was removed. Factor 4, designated distance awareness, was composed of items 1 and 3, and accounts for 7.2% of the variability. All loadings were significant and above 0.30, and were retained as the factor was defined solely by appropriate marker items.

*Step 4: Confirmatory Factor Analysis*. To evaluate the quality of adjustment of the measurement model and further corroborate the LAHCS factor structure, using Mplus software, an additional EFA was performed on the remaining 13 items and on sub-sample 1 (*N_subsample_*_1_ = 746). As shown in Table 3, the four-factor model provided an acceptable fit to the data (χ^2^ [32] = 170.47, SRMR = 0.02, RMSEA = 0.08, CFI = 0.96; Hu & Bentler, 1998) and fit the data significantly better than any other alternative models. Each item loading on the intended factor was substantial and significant.

Based on the results from EFA, we proceeded to CFA on sub-sample 2 (*N_subsample_*_2_ = 692). The CFA of the LAHCS indicated that the 4-factor model had an acceptable fit to the data (χ^2^ [59] = 264.91, SRMR = 0.05, RMSEA = 0.07, CFI = 0.93; Hu & Bentler, 1998; see Table 1). Overall, EFA and CFA supported the hypothesized four-factor structure defined by 13 items of the LAHCS, providing evidence of factorial validity.

Table 4 shows the descriptive statistics, reliabilities and correlations among the four dimensions of the LAHCS for the first (*N_subsample_*_1_ = 746) and second (*N_subsample_*_2_ = 692) subsamples, performed using the final factor structure. The obtained Cronbach’s alpha coefficients were robust (i.e., above 0.80; Konting et al., 2009) and ranged from 0.81 to 0.90 for the four LAHCS subdimensions. Moreover, the total-item correlation coefficients for the four subdimensions ranged between 0.51 and 0.82. In particular, alpha coefficients of the two 2-item subdimensions (i.e., distance awareness, behavior change) were good (>0.80), thereby corroborating the appropriateness of the two-item structure to measure the focal variables (Eisinga et al., 2013). Overall, the item analysis provided additional support for the final 13-item structure of the LAHCS (see Appendix A for the final structure).

*Step 5: Convergent/Discriminant validity*. Next, before proceeding to test discriminant validity, using the total sample (*N_total_* = 1454), we preliminarily assessed the construct validity of other leadership scales and performed a CFA to demonstrate the appropriateness of the seven hypothesized latent factors (i.e., transformational, transactional, leader group prototypicality, leader–member exchange, authentic, and servant leadership styles, and group member prototypicality). As shown in Table 3, the CFA of other leadership scales and GMP had good fit indices (χ^2^ [2238] = 8193.61, SRMR = 0.05, RMSEA = 0.04, CFI = 0.91), thus supporting the distinctiveness among the study variables.

We then conducted CFAs to test the discriminant validity of the LAHCS (i.e., a leadership variable) from GMP (i.e., a non-leadership variable) on the total sample (*N_total_* = 1454). The benchmark model has five factors treating the four dimensions of the LAHCS and GMP as five separate concepts. In addition to the benchmark model, we tested five alternative models, including four four-factor models and one one-factor model. As can be seen in Table 3, Model 1 treated emotional exchange awareness and GMP as a single factor, Model 2 treated exploration and GMP as a single factor, Model 3 treated distance awareness and GMP as a single factor, and Model 4 treated behavior change and GMP as a single factor. Model 5 had only one factor, where four dimensions of the LAHCS were combined with GMP as a single factor. As shown in Table 2, the five-factor benchmark model fit the data well (χ^2^ [125] = 903.20, SRMR = 0.05, RMSEA = 0.07, CFI = 0.91) and was significantly better than the five alternative models. As such, the five-factor model provided evidence for the discriminant validity of the LAHCS in terms of conceptual distinctiveness from non-leadership variables (i.e., GMP).

To demonstrate the convergent and discriminant validity of the LAHCS, we used disattenuated correlation coefficients performed on the total sample (*N_total_* = 1454). Table 5 shows the statistically significant disattenuated correlation between dimensions of the LAHCS and other leadership scales (i.e., convergent validity). Specifically, awareness of holistic emotional exchanges had positive correlations with all other leadership scales, ranging from 0.30 to 0.48. Similarly, exploration had positive correlations with all other leadership scales, ranging from 0.28 to 0.39. Behavior changes had positive correlations with all other leadership scales, ranging from 0.49 to 0.57, whereas distance awareness had negative correlations with all other leadership scales, ranging from −0.21 to −0.31. Moreover, the four LAHCS’ dimensions displayed statistically significant positive disattenuated correlations with GMP (i.e., discriminant validity), ranging from 0.12 to 0.26 (i.e., the lowest correlations indicating poor to negligible links), with the exception of distance awareness, which was not significant. Taken together, the LAHCS showed good convergent validity with other leadership constructs and discriminant validity from a non-leadership construct.

## 4. Study 2: Nomological, Incremental, and Cross-Cultural Validity

Following the validation steps recommended by Hinkin (1998, i.e., Step 6: replication), Study 2 was conducted to provide data on the measurement invariance of the Italian and English versions of the LAHCS across Italian and American samples of employees to determine if members of different cultures interpret the measure’s items in a conceptually similar way (Deffner et al., 2022). Moreover, measurement invariance was the premise to the subsequent structural equation model testing a nomological network aimed at assessing the criterion validity of the LAHCS dimensions in predicting employees’ well-being, supervisor satisfaction and organizational commitment (i.e., nomological validity; F. Kock et al., 2024). The theoretical basis for how the LAHCS dimension fits within a network of well-being and job attitude constructs examined as relevant leadership outcomes (i.e., criteria) was provided in the above literature review, along with Hypotheses 1 and 2 regarding the links within the nomological network. Finally, the study assessed whether the LAHCS has a unique explanatory power of outcome variables (i.e., burnout, commitment and satisfaction) and demonstrates incremental validity beyond extant leadership-related constructs.

### 4.1. Method

#### 4.1.1. Sample and Procedure

The procedure of the Italian data collection was the same as described in Study 1.

The Italian sample included 188 employees from various organizations. Sixty-eight percent of participants were male, and 31.9% were female. The ages ranged from 18 to 67 years, with a mean age of 28.60 years (*SD* = 9.92). Sixty-six percent of participants completed high school, 24.1% college or higher, and 10.2% junior high school. The mean organizational tenure was 7.3 years (*SD* = 6.92) and ranged from 1 to 35 years. Seventy-five percent of the sample worked for a private company, and the majority of the participants (89.9%) held a non-managerial position. Regarding the contract type, 65.9% of the sample had a temporary position, and 71.4% of participants worked full-time. While the questionnaire did not include items on the organization type, the respondents who agreed to participate in the study belonged to organizations in the occupational areas of mail services, construction, public administration, sport, and the military.

For the American sample, participants included 400 employed individuals who responded to an online survey in exchange for a $1 payment. Participants were recruited through American’s Mechanical Turk, which is argued to be a reliable data source (Buhrmester et al., 2011). As such, the anonymous survey data were collected cross-sectionally at a single point in time from a sample of American adult workers. The sampling technique was based on a snowball convenience sample strategy. Thirty-three individuals failed attention checks and were removed from analyses. Of the remaining 367 respondents, 63.5% of the sample were female, 52.9% worked in the public sector; 42% held a management position; 92.9% had an open-ended contract; 84.2% were full-time employees, and 40.3% had a 4-year degree. On average, participants were 35.99 years old (*SD* = 11.90). Organizations belonged to the following occupational sectors: retail (12.8%); hospitality (6%); health care (15.3%); education (12.3%); manufacturing (3.8%); transportation (2.7%); communication and technology (13.9%); military (1%); artistic (2.5%) construction (4.4%); services and finance (11.2%), and approximately thirteen percent did not specify the sector.

#### 4.1.2. Transparency and Openness

The covariance matrix and analysis code are available upon request from the first Author. Row data for this study are not available as we do not have permission from participants for row data sharing.

### 4.2. Measures

The US and Italian versions of the survey contained the following scales, respectively worded in English and Italian. Items from the LAHCS were translated into English from the Italian version, and items of supervisor satisfaction and affective commitment were translated into Italian from the English version using the standard translation-back-translation procedure recommended by Brislin (1980). The correspondence of the original and the back-translated items was then verified by the authors.

**Leader Awareness of Holistic Contagion Scale.** The version of the scale is the final 13-item version as previously described in Study 1 (see Appendix A).

**Job Burnout.** Five items of the exhaustion subscale of the Maslach Burnout Inventory—General Survey (MBI—GS; Schaufeli et al., 1996) measured employees’ emotional and physical depletion associated with a lack of energy to face work situations, and five items of the cynicism subscale measured employees’ feelings of callous detachment from their work. A sample exhaustion item was “*I feel emotionally drained from my work*”, and a sample cynicism item was “*I am becoming more detached from my work*”. Items were rated on a 7-point frequency scale ranging from *never* (0) to *daily* (6).

**Supervisor Satisfaction.** Employees’ satisfaction with their supervisor was measured using nine items from the satisfaction with supervisor subscale of the Job Descriptive Index (Smith et al., 1969). A sample item was “*My supervisor is tactful*”. Respondents indicated on a three-point scale (*yes, don’t know,* or *no*) the extent to which each adjective or phrase described the stability of their job. Item responses were coded as follows: agreement with positively worded items (e.g., “*Praises good work*”) was scored 3; agreement with negatively worded items (e.g., “*Impolite*”) was scored 0; and “don’t know” responses were scored 1. As such, negatively worded items were reverse-coded such that higher scores of the scale reflected greater supervisor satisfaction.

**Affective Commitment.** Affective organizational commitment was measured using eight items from Allen and Meyer’s (1990) Affective Commitment Scale. A sample item was “*I enjoy discussing my organization with people outside it*”. Negatively worded items were reverse-coded such that higher scores of the scale reflected greater affective commitment. Items were rated on a seven-point agreement scale ranging from *strongly disagree* (1) to *strongly agree* (7).

**Leadership Scales.** The leadership scales were the same as described in Study 1 (transformational leadership, transactional leadership, leader group prototypicality, leader–member exchange, authentic leadership, and servant leadership), respectively worded in English and Italian.

### 4.3. Analytical Strategy

To follow Hinkin’s (1998) recommendations on a scale’s development (i.e., Step 6: replication) and additionally test the LAHCS’ criterion (nomological) validity, the following analyses were performed. We used Meredith’s (1993) framework to examine measurement invariance, including four steps. First, *configural invariance* examines whether a construct has the same meaning and basic factorial structure in two different groups. In the current study, configural invariance would confirm that the latent constructs of the LAHCS were similarly reflected in either culture, as the observed items would load on the latent variables as hypothesized. Second, by constraining factor loadings to be equal across groups, *metric invariance* confirms that the factor loadings of individual items on respective factors are equal in two groups. Third, by constraining both factor loadings and intercepts of indicators to be equal across groups, *scalar invariance* confirms that the scoring of the latent construct (item factor loadings) and the level of scale origin (item intercept and means) are equivalent across two groups. Last, by constraining factor loadings, intercepts, and residual variances to be equal, *strict invariance* suggests that the same score of a latent variable across different groups corresponds to the same conditional variability of its observed score. Usually, CFI differences (ΔCFI) are used to evaluate measurement invariance. CFI differences no larger than 0.01 are usually employed as the cut-off criteria (Cheung & Rensvold, 2002). If all constraints are tenable, one can claim complete metric, scalar, or strict invariance, allowing for a comparison of regression coefficients linking latent variables within a full structural equation model. Once ME is supported at least at the level of metric invariance, we can test if the structural model is equivalent across the two countries, i.e., if the path coefficients linking the constructs in the hypothesized nomological network are the same in the U.S. and in Italy.

Finally, in order to assess whether the LAHCS explains incremental variance of the current research’s criterion variables beyond extant leadership-related constructs, we performed on both the Italian (*N_Italy_* = 188) and U.S. (*N_US_* = 400) sample a set of hierarchical regression analyses. Specifically, we performed three regressions, respectively, positing job burnout, affective commitment, and satisfaction with supervisor as the outcome variable. We built the regression model by entering at step 1 as independent variables the leadership-related constructs altogether (i.e., transformational leadership, transactional leadership, leader group prototypicality, leader–member exchange, authentic leadership, and servant leadership). Next, we entered at step 2 the LAHCS total score and assessed the change in R-squared (i.e., the proportion of variance explained). A significant ΔR^2^ would suggest that the added variable contributes unique explanatory power to the model, beyond what is already explained by the other leadership-related predictors.

### 4.4. Results

Table 6 shows the descriptive statistics, reliabilities, and correlations among the variables for the Italian and American samples.

#### 4.4.1. Measurement Invariance Across Two Countries

Prior to conducting multiple-group invariance analyses, we examined the goodness-of-fit values for the CFA models for Italian and American samples separately. The values for Italian samples were χ^2^(59) = 71.59, RMSEA = 0.05, CFI = 0.97, SRMR = 0.06, showing a good fit to the data. Similarly, the values for the American sample were χ^2^(59) = 146.69, RMSEA = 0.06, CFI = 0.96, SRMR = 0.04, indicating an acceptable to good fit. Table 7 also shows the results of analyses for invariance testing. Each of the four invariance models provided a good fit, and the decrease in the CFI value was no larger than 0.01 for each invariance comparison. As such, there was good evidence for the equality of forms, loadings, intercepts, and residuals across the two countries.

#### 4.4.2. Structural Model for Hypothesis Testing

Before examining the nomological/criterion validity of four dimensions of the LAHCS, we created item parcels for construct measures with more than three items for the Italian sample because of its relatively small sample size (Little et al., 2002). Item parcels were created by sequentially assigning items per parcel based on the highest to lowest item-to-construct loadings/correlations (Little et al., 2002). We examined the goodness-of-fit index for the structural equation models using four dimensions of the LAHCS as predictors and exhaustion, cynicism, supervisor satisfaction, and affective commitment as outcomes in the Italian and American samples separately. Given the parcelled data of the Italian sample, we did not perform structural invariance and tested the nomological networks separately for Italy and the U.S. The structural equation model fit the data well in both Italian (χ^2^ [247] = 365.50, SRMR = 0.06, RMSEA = 0.051 [0.039–0.061], CFI = 0.94) and American (χ^2^ [712] = 1392.10, SRMR = 0.05, RMSEA = 0.051 [0.047–0.055], CFI = 0.92) samples.

**Figure 1:**
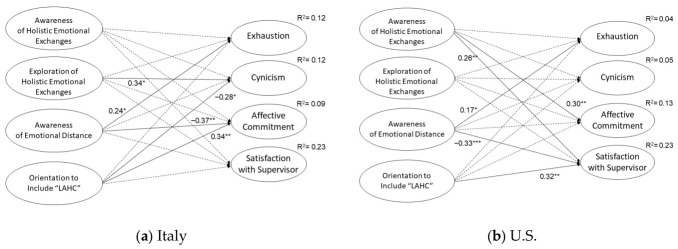
Standardized structural coefficients for the final structural model, respectively, for Italy and the U.S. Dotted lines are non-significant effects. * *p* < 0.05; ** *p* < 0.01; *** *p* < 0.001.

As can be seen in Figure 1a, in the Italian sample, exploration was positively related to cynicism (0.34, *p* < 0.05). Emotional exchange awareness was not significantly related to any of the outcomes. Distance awareness was positively related to exhaustion (0.24, *p* < 0.05) but negatively related to affective commitment (−0.37, *p* < 0.01). Behavioral change was negatively associated with cynicism (−0.28, *p* < 0.05), but positively related to affective commitment (0.34, *p* < 0.01). Finally, the model explained 12% of the variance in exhaustion, 12% in cynicism, 9% in satisfaction with supervisor, and 23% in organizational commitment. Overall, results partially supported Hypothesis 1a and 1b as well as Hypothesis 2b.

In the American sample (Figure 1b), emotional exchange awareness was positively related to supervisor satisfaction (0.26, *p* < 0.01) and affective commitment (0.30, *p* < 0.01). Exploration was not significantly related to any of the outcomes. Distance awareness was positively related to exhaustion (0.17, *p* < 0.05), but negatively related to supervisor satisfaction (−0.33, *p* < 0.001). Behavioral change was positively associated with supervisor satisfaction (0.32, *p* < 0.01). Finally, the model explained 4% of the variance in exhaustion, 5% in cynicism, 23% in satisfaction with supervisor, and 13% in organizational commitment. Overall, results partially supported Hypothesis 1a as well as Hypotheses 2a and 2b.

#### 4.4.3. Incremental Validity

Before proceeding to incremental validity assessment, we examined the descriptive statistics and reliabilities of the variables included in the analyses (i.e., burnout, commitment, satisfaction with supervisor, LAHCS, transformational, transactional, LMX, authentic, servant leadership, and LGP). For the sake of parsimony, we considered the total score of job burnout (i.e., the mean of exhaustion and cynicism subdimensions) and the LAHCS total score (i.e., the mean of the four subdimensions). Results are reported in Table S1 of the Supplementary Materials.

Table 8 shows the results of the three hierarchical regression analyses performed on the Italian (*N_Italy_* = 188) and U.S. (*N_US_* = 400) samples, respectively, positing job burnout, affective commitment, and satisfaction with supervisor as the dependent variables and the leadership-related constructs, as well as the LAHCS score, as independent variables. As can be seen, all three regressions in both the Italian and U.S. samples showed a statistically significant increase in R-squared in burnout, affective commitment and satisfaction with supervisor when adding the LAHCS score (with the exception of affective commitment in the U.S. sample). Overall, the results showed that adding the LAHCS significantly increases the percentage of variance explained in burnout, commitment, and satisfaction after controlling for other relevant leadership-related predictors, thus supporting the notion that the LAHCS has a unique explanatory power and demonstrates incremental validity beyond extant related constructs.

## 5. Discussion

The majority of the existing leadership literature acknowledges the importance of emotion management for effective leadership action (e.g., Antonopoulou, 2024; Halimah et al., 2014). Yet, only a few leadership theories (e.g., servant and transformational) embed emotion management within their theorizing and accompanying operationalization, and no theory does it from the perspective of contextual emotions that involuntarily spread among multiple stakeholders at work (i.e., holistic emotional contagion). To fill this gap, the present research engaged an inter-personal approach to the emotional processes intersecting leadership and aimed to complement existing leadership models by proposing a conceptual framework that embeds a leader’s mastery of contextual contagion dynamics among multiple organizational stakeholders (e.g., colleagues and clients). Specifically, the research aims to: (1) include emotional contagion in leadership theorizing and assess the psychometric properties of the newly developed accompanying scale (i.e., LAHCS), (2) assess the construct, convergent, discriminant and cross-country (i.e., Italy and US) validity of the LAHCS, and (3) examine the extent to which the four dimensions of the contagion leadership model (i.e., LAHC) predict employees’ well-being (i.e., burnout) and attitudes (i.e., satisfaction with supervisor and organizational commitment).

Our findings from two cross-sectional and cross-country (i.e., Italy and U.S.) studies provided support for the internal, convergent and discriminant validity as well as measurement invariance, nomological and incremental validity of the four-dimension LAHCS (i.e., leader awareness of holistic emotional exchanges, leader–follower(s) emotional distance awareness, exploration of holistic emotional exchanges, and willingness to include “LAHC” model) across the English and Italian versions. In addition to demonstrating nomological/criterion validity, the LAHCS also explains incremental variance in workplace outcomes (i.e., burnout, commitment, and satisfaction with supervisor) beyond extant leadership-related constructs, thus supporting the relevance of this new construct and contributing to the knowledge in the literature on leadership and emotions. Moreover, our results suggest that a leader’s awareness of holistic contagion and/or their orientation to manage contagion is associated with higher followers’ commitment and satisfaction with the supervisor. More importantly, the leader’s engagement in the active exploration of these contagion exchanges and their awareness of the emotional distance that their followers experience towards him/her is associated with higher followers’ burnout and lower commitment and supervisor satisfaction. Overall, our cross-country findings reveal that while followers appreciate a leader’s awareness of emotional traffic, attempts to actively explore this traffic or being aware of the followers’ emotional distance can create discomfort, resulting in dissatisfaction with the leader, decreased organizational commitment, and poor well-being. This may occur because the leader’s behaviors are perceived as intrusive or embarrassing. Below, we delve into the discussion of these counterintuitive findings.

### 5.1. Theoretical Implications

Our findings have implications for the extant literature in the areas of leadership, emotional contagion and occupational health. First, our research adds to the leadership literature and complements existing models by examining a leader’s action, embedding a mastery of contagion dynamics (i.e., LAHC model) and proposing a conceptual framework that shifts the focus of leadership emotional processes from more common inter-individual approaches (e.g., EI and empathy) towards an inter-personal and more expanded perspective of emotions exchanged among multiple stakeholders in the workplace (i.e., leaders, colleagues, and clients). Notably, given the intrinsic inter-personal nature of emotional contagion that allows an awareness of how one’s emotions are absorbed and experienced by others, and the reverse (Hatfield et al., 1994; LeDoux, 2002; Petitta & Naughton, 2015), the LAHC framework qualifies as an emotion–leadership model grounded in a leader’s simultaneous awareness of both their feelings and those of others. That is, a personal as well as a situational emotion awareness leadership process. Overall, the LAHCS is a valid and reliable measure with unique explanatory power of workplace outcomes above and beyond extant leadership models, thus demonstrating the added value of this new construct. Moreover, while the literature tends to recommend pre-test content validity assessment of newly developed items (e.g., Cortina et al., 2020; Colquitt et al., 2019), this step is suggested in order to “minimize” later item deletion due to poor items’ clarity and understanding for a reader (Hinkin, 1998). In the current research, we reached this goal of checking content clarity for readers also using cross-country measurement invariance tests and demonstrated items “content adequacy” (i.e., Schriesheim et al., 1993) by showing that the LAHCS questions display no item bias and appear to have the same meaning for both Italian and American readers or rather, semantic equivalence in different languages and overall content validity of the LAHCS’ statements. Moreover, while the four LAHCS sub-dimensions may appear conceptually similar and overlapping, our CFAs’ results comparing alternative multiple-factor solutions supported the conceptual distinctiveness among the four facets, thus suggesting that their similarity is likely due to the call into account of the common underlying concept of holistic contagion exchanges.

Our results from the study of the LAHCS’ psychometric properties support its convergence with other leadership models and difference from non-leadership constructs (i.e., group member prototypicality). Moreover, our findings advance leadership theory by placing leader–member emotional and relational dynamics within the framework of a context-specific and comprehensive mapping of emotional exchanges among all key stakeholders at work. Interestingly, willingness to include the LAHC model displays the highest correlations with all leadership models. That is, a leader’s orientation to change and include this additional contagion-oriented perspective is associated with all relationship-focused leadership styles (e.g., Dinh et al., 2014) considered in the current research. More interestingly, a leader’s awareness of emotional distance from their followers negatively correlates with all leadership styles. That is, a leader who is aware that their followers share more confidences among themselves rather than with him/her and feel emotionally more distant from him/her in comparison to their peers is perceived as less prototypical of their group and less intrinsically concerned with social transactions with followers and their needs as well as their growth (e.g., LGP, LMX, transactional, and servant leadership styles). This counterintuitive finding is consistent with the criterion validity results, showing that this LAHC’s dimension also predicts higher follower distress and dissatisfaction.

An arguable explanation of the negative link between leaders’ awareness of emotional distance from their followers and employees’ poor well-being and satisfaction may call into account a potential lack of psychological safety (i.e., the belief that it is safe to speak up; Edmondson & Mortensen, 2021). That is, when employees feel unable to ask for help or share concerns about work due to leader–follower distance, this likely increases the risk of feeling overwhelmed and experiencing burnout (Kerrissey et al., 2022). An additional explanation may rely on emotional labor dynamics, such as surface acting (modifying expressions) and deep acting (modifying moods) strategies of employees (Sayre et al., 2025), in order to face leaders’ awareness of emotional distance, which may wear out their energies. A closer explanation of the contagion literature suggests that followers are spontaneously more attracted and emotionally closer to each other (e.g., Petitta et al., 2023), making this explicit within the leader–follower(s) relationship seems to create unwanted leadership consequences. Indeed, the literature on emotional processes (e.g., EI) increasingly points to some potential dark sides of these factors and suggests the existence of the too-much-of-a-good-thing effect such that emotionally sensitive and intelligent people who are aware of those around them may tend to shy away from typical leadership tasks, such as making decisions or providing negative feedback that might be unpopular (e.g., Blaszczak-Boxe, 2017; Grant, 2014; Lubbadeh, 2020), thus hampering the guiding and directing actions expected by efficacious leaders (Kerr et al., 2006). Similarly, one may argue that while followers expect leaders to provide them with guidance, respect and understanding (e.g., Kelley, 1988; Kerr et al., 2006), they may feel uncomfortable if the leader gets overly familiar and emotionally too close (e.g., Burleson, 2022; Chand, 2019; Flaxington, 2013). Overall, our findings suggest that a follower who perceives their leader as being aware of their emotional distance may allude to an elephant in the room within the leader–member relationship and thus, contribute to unraveling an additional dark side of emotional processes associated with leadership.

Second, our research also bridges emotional contagion and leadership studies. We extended earlier studies on the contagion leadership investigation that has mainly focused on downward (i.e., how leaders transfer their emotions to followers; Bono & Ilies, 2006) and upward (i.e., how followers infect their emotions into the leader; Dasborough et al., 2009) contagion and considered emotional contagion as a separate mechanism from leadership. The LAHC model, cross-culturally validated in the present study, advances the emotional contagion literature by proposing an inter-personal and more expanded perspective of emotions exchanged among multiple stakeholders in the workplace (i.e., leaders, colleagues, and clients). In so doing, the new model integrates into leadership theorizing the differential contribution of people holding different roles to emotional dynamics at work and the extent to which the leader masters these holistic emotion-spreading mechanisms in the workplace. As such, the LAHC model overcomes the drawback of examining the leader–follower(s) dyad in isolation and shows how a relation-based framework with several foci may help to recognize that emotional ambience is co-created by all parties involved in the relationship (Arizmendi, 2011) and improves our understanding of the true emotional bonds between leaders and followers. Relatedly, our finding that a leader’s awareness of emotional distance from their followers predicts higher follower distress, dissatisfaction and lack of commitment also brings to the forefront the effects of a leader’s (lack of) emotional primacy within the emotional orbit of their followers on employees’ attitudes and well-being. On the one hand, a leader’s minimal emotional involvement with their followers (as compared to other stakeholders) should not be undervalued, and leaders should be mindful of their positioning within followers’ emotional orbit. On the other hand, emotional orbit and power orbit should be differentiated, and a leader’s lack of emotional primacy does not negate the power dynamics and social influence inherent in leadership acts (Fisk & Friesen, 2012; Kelman, 1958). Yet, future studies should examine how they intersect and the extent to which they overlap when assessing the leader’s grip on their followers.

Third, our findings add to organizational and health psychology by unveiling some counterintuitive and unexpected consequences of emotions and leadership processes. Specifically, we extended earlier research on leadership’s effects on employees’ distress (e.g., Harms et al., 2016) and job attitudes (e.g., Hancock et al., 2023; Ismail et al., 2010) by showing a relationship between leadership factors grounded in the mastery of contagion dynamics and followers’ burnout, satisfaction with their leader and organizational commitment. Notably, the four LAHC’s dimensions exert apparently contradicting effects on followers’ health and attitude outcomes. According to our findings, a leader who is aware of emotional traffic among different roles and who is willing to manage these complex contagion dynamics makes their followers more satisfied with their supervision and more committed to the organization. However, a leader who is interested in and actively examines with the followers how comfortable they feel with him/her or the reasons why they share more confidence among themselves rather than with him/her and feel emotionally distant from the leader makes employees exhausted, detached, dissatisfied with their leader and uncommitted towards the organization. While the results are slightly different in the U.S. and Italian samples, the overall pattern of associations among these variables is consistent across the two different national settings. One potential post hoc explanation of the composite pattern of results revealed by the contagion leadership model (i.e., LAHC) may rely on the existence of a non-linear quantitative relationship due to the potential curvilinear effects of leadership factors on employees’ outcomes (e.g., too-much-of-a-good-thing effect), as is the case with other emotion-related within-person constructs like EI (e.g., Lubbadeh, 2020). However, qualitative effects due to the diverse inter-personal and contextual emotions management dimensions conceptualized by the LAHC seem more likely. That is, across different cultural contexts, a leader who is mindful of, and is willing to manage, complex contextual emotional processes is appreciated, but the leader’s attempt to explicitly face and examine the emotional magnets that attract followers among themselves and marginalize the leader likely engenders a social discomfort that results in followers’ dissatisfaction and poor commitment and well-being. Overall, the contagion leadership model may serve as a springboard to the understanding of some apparently inconvenient findings about emotions and leadership and their links to employees’ health and job attitudes, thus advancing occupational and health psychology studies by broadening the scope of leadership’s action.

### 5.2. Implications for Practice

The findings from the current research have several implications for practice. Indeed, our cross-country results show how different facets of the leader’s mastery of contagion dynamics may diversely and oppositely associate with their followers’ well-being, satisfaction and commitment, and suggest two main areas of organizational intervention.

First, the LAHC model offers a different approach to the study of emotions in relation to leadership and points out that in organizational settings leaders and followers are not isolated but share the working context with multiple stakeholders with whom they simultaneously interact, and allows an awareness of emotions that are exchanged not only between leaders and collaborators, while also inviting leaders to interact with their followers by actively managing emotional exchanges in the context. Organizations are advised to increase training for leaders to help them recognize and understand that emotional ambience is co-created by all parties involved in the relationship (Arizmendi, 2011) and become more conscious of their emotional position in relation to followers and clients. Here, the newly developed LAHC scale may assist in at least two ways. On the one hand, followers’ hetero assessment of the leader through the four LAHCS dimensions may provide feedback on the leader’s awareness and mastery of contagion dynamics. On the other hand, understanding how emotions propagate should help leaders get familiar with emotional mirroring (e.g., Sowden et al., 2022) in the workplace, thus grasping and proactively managing both their own and others’ emotions beyond the person-centered perspective of their empathy or EI.

Second, leaders are the key to influencing other people in order to drive and facilitate conjoint working activities, and leadership is the fundamental driving force of organizational success (Yukl, 2010). Yet, our composite findings on the effects of the LAHC’s leadership factors may appear challenging to reconcile with this statement and tricky to disentangle, given that a leader who is mindful of complex contextual emotional processes enhances followers’ well-being and satisfaction, but their attempt to actively examine the emotional magnets that attract followers among themselves and marginalize the leader ends up resulting in follower dissatisfaction and distress. Indeed, when leaders avoid addressing uncomfortable and unspoken issues, such as subtle team conflicts, those problems only fester and likely generate psychological strain and further suppressed voices over time. Conversely, leaders’ engagement in bringing hidden issues and unspoken fears or hesitations to the surface in order to transparently address them with followers may help in demonstrating leader’s caring of team sentiment and wealth, thus creating a safe and comfortable environment to speak up and solve task or relational problems (e.g., Hubbart, 2024; Yin et al., 2022). As such, our provoking finding on the potential negative consequences of leaders’ awareness of their marginal emotional role may help in bringing to the forefront the relevance of implementing leadership action in organizations that proactively engage in unfolding and overcoming emotion-related issues (e.g., leader emotional marginality), rather than stagnating into worries of resulting intrusive and avoiding the “elephant in the room”. The holistic mapping of the LAHCS may assist in developing interventions aimed at constructively using both the bright and dark sides of the contagion leadership, if we adopt an agentic perspective of individuals learning from experience and being able to actively manage their social context (Bandura, 1986). On the one side, the literature on social processes such as intimacy and embarrassing situations suggests that uncomfortable social circumstances serve adaptive purposes of expressing an emotion that helps to repair social relations, elicits forgiveness, and advertises positive character traits (i.e., trustworthiness), thus bridging social bonding that enhances the sense of ingroup (e.g., Eller et al., 2011). As such, uncomfortable conclusions in the leader–follower relation may be worth facing rather than being left unsaid. On the other side, training interventions may give leaders feedback on the outcomes of contagion leadership mapping, make them aware of the systematic emotional primacy of coworkers in the workplace, and enable leaders to use the LAHC’s profile results to manage the emotions that develop between a leader and their followers, as well as actively examine, grasp, and proactively manage the emotional interactions among followers themselves. Towards this end, the practical group space model (Bachmann, 2022) may guide leader training in facing typically problematic social knots in team dynamics, such as *hierarchy* (i.e., power), *affiliation* and *intimacy*. Examining affiliation-related issues may help leaders become more aware of frictions caused by “ingroup or outgroup” feelings and relational dynamics, while training on hierarchy issues enables one to learn about and become self-aware of tensions arising from “top or bottom” aspects in a social context. Last but not least, intimacy/familiarity issues enable group members to regulate their proximity and distance from one another as well as recognize the tensions brought on by “near or far” problems in social interactions. Together, providing leaders with training that combined with the LAHC’s mapping results may cover the intricacies of handling bonding and cohesion in organizational settings that underpin effective leadership action. Moreover, given the intrinsic multi-stakeholder nature of the LAHC model, interventions aiming to promote leaders’ effective management of contagion dynamics in order to foster employees’ commitment and well-being may target multiple levels of the stratified influence of leadership in organizations (i.e., supervisors, top management; Petitta & Borgogni, 2011).

### 5.3. Strengths, Limitations and Future Directions

Despite the strength of unfolding how different facets of the leader’s mastery of contagion dynamics diversely and oppositely associate with their followers’ well-being, satisfaction and commitment, the current research also suffers from some limitations. The first limitation of this research is that all variables were measured through self-report, which introduces the potential for common method variance associated with single-source data collection (Podsakoff et al., 2003). However, self-reports are essential for assessing employees’ burnout and job attitudes, including supervisor satisfaction and affective commitment. Moreover, the disattenuated correlations among the study variables showed the simultaneous existence of high and low correlations, thus suggesting that common method bias is unlikely to have occurred in our study (Spector, 2006). Nonetheless, future research could address this limitation by incorporating assessments from alternative sources (e.g., leaders) to mitigate common method bias.

A second limitation is the use of cross-sectional survey data, which are particularly problematic in Study 2 when assessing criterion validity. This design prevents us from drawing causal conclusions about the relationships between LAHC and employee burnout, supervisor satisfaction and affective commitment. It is also plausible that employees with high supervisor satisfaction are more likely to report higher levels of LAHC in line with the literature (e.g., Maleka, 2024), suggesting a reciprocal relationship, such that a positive emotional leadership perception increases a “happier” workplace, which in turn may encourage a more favorable leadership perception. Future research should adopt a longitudinal approach to better evaluate the predictive power of LAHC over time. Causality concerns could be mitigated through the use of two-wave data that introduce temporal distance between our study variables in order to assess whether concomitant increases or decreases in the LAHC dimensions are associated with similar trends in outcome variables such as employee burnout, commitment and satisfaction with leadership.

An additional strength of the current research is the cross-country data (and proven measurement invariance) from the U.S. and Italy that allows generalizing our findings to different cultural contexts. Future research may help to further extend the ecological validity (Deffner et al., 2022) of our results by testing the hypothesized nomological network in different national contexts other than European and North American ones.

Finally, future studies might also extend the current findings by examining other leadership outcomes such as leadership effectiveness (e.g., Madanchian et al., 2017). Moreover, results on the leader’s awareness of emotional distance from followers allow for disentangling emotional orbit from power orbit in a leader’s action. Yet, future research should further examine the incremental effects of power factors (e.g., Nesler et al., 1999) on leadership effectiveness above and beyond the emotional orbit captured by the LAHC’s dimensions.

## 6. Conclusions

The present study is the first to (1) include the leader’s mastery of holistic emotional contagion (i.e., emotions that involuntarily spread among multiple stakeholders at work) into leadership theorizing, (2) assess the cross-country validity of the accompanying measure (Leader Awareness of Holistic Contagion Scale: LAHCS), and (3) examine the LAHCS’ convergent, discriminant and nomological/criterion validity using common leadership models in the existing literature (i.e., leader group prototypicality, leader–member exchange, authentic, transactional, transformational, and servant leadership). The multi-study and cross-country research design provided support for the measurement invariance, validity and reliability of the newly developed LAHC scale, including the four facets of leader awareness of holistic emotional exchanges, exploration of holistic emotional exchanges, leader–follower(s) emotional distance awareness, and willingness to include “LAHC”. Noteworthily, our findings also reveal some unchartered truths about leadership and emotions, such that leaders who are aware of workplace emotional traffic (e.g., awareness of holistic contagion and orientation to manage contagion) are appreciated, but if they attempt to explore this traffic actively or are aware of followers’ emotional distance, then the situation becomes likely intrusive, uncomfortable and results in followers’ dissatisfaction, poor commitment and higher burnout. For scholars and practitioners alike, our findings provide a conceptual framework underpinning leaders’ management of contextual emotions at work (i.e., involuntarily spreading among multiple stakeholders that surround the leader–member dyad), which complements more common person-centered emotional processes (e.g., emotional intelligence).

## Figures and Tables

**Table 1:** Conceptual comparison among emotional contagion, empathy and emotional intelligence.

	Emotional Contagion	Empathy	Emotional Intelligence
*Conceptual Core*	Transfer of emotions among individuals	Sensing and understanding others’ emotions	Managing (using) of emotions (owns and others’)
*Focus*	Involuntary absorptions of others’ emotions	Voluntary understanding	Voluntary using
*Mechanisms*	Mimicry Feedback Mirror neurons	Sensory–motor resonance Affective sharing Mental state perspective-taking Mirror neurons	Sensory–motor resonance Affective sharing Mental state perspective-taking Mirror neurons
*Type of Construct*	Pre-reflective (automatic) resonance	Skill/disposition	Skill/disposition
*Perspective*	Relation-centered	Person-centered	Person-centered
*Level*	INTER-personal	INTRA-Individual	INTRA-Individual
*Key Conceptual Features*	Precursor of empathy	Builds on contagion	Includes empathy
*Questions answered*	Whom did I get this emotion from?	Am I understanding how the other is feeling?	How can I use this emotion?

**Table 2:** Conceptual comparison between the LAHC model and common relational/emotional leadership models (LMX, servant leadership, and transformational leadership).

	LAHC	LMX	Servant Leadership	Transformational Leadership
*Stakeholders*	Leader Followers Clients/Externals	Leader Followers	Leader Followers	Leader Followers
*Conceptual Core*	Leaders’ awareness of and willingness to manage holistic emotional exchanges among different roles in their own context (i.e., multiple stakeholders such as leaders, colleagues and clients)	Leader providing support, autonomy, affection, and responsibility to collaborators in exchange for their commitment	Leader at the service of his/her followers by emotionally supporting them and prioritizing their emotional well-being	Leader passionately inspires followers towards a common goal and motivates them to achieve it
*Focus*	Contextual dynamics of emotional exchanges beyond the leader–follower dyad	Leader–follower relationship	Followers’ needs/emotions	Follower’s motivation
*Key features*	Quality of emotional exchanges (all stakeholders)	Quality of relations (leader–follower)	Emotional support	Motivational support
*Emotion* *Embeddedness*	Emotion-related mechanisms (i.e., emotional contagion) *intrinsically embedded* in the leadership model	Emotion-related mechanisms *not* intrinsically *embedded* in the leadership model	Emotion-related mechanisms (e.g., empathy) *partially considered* in the leadership model	Emotion-related mechanisms *not* intrinsically *embedded* in the leadership model
*Perspective*	Relation-centered	Relation-centered	Person-centered	Person-centered
*Level*	INTER-personal	INTER-personal	INTRA-Individual	INTRA-Individual
*Questions Answered*	Whom did the followers get this emotion from? Me? Clients? Teammates? Where did this emotional ambience come from?	How is the quality of my relationship with the followers?	Am I emotionally supporting the followers?	Am I motivating and supporting followers toward common goals?

**Table 3:** Model fit indices from exploratory and confirmatory factor analysis in Study 1.

	*N*	*df*	χ^2^	χ^2^/*df*	CFI	TLI	RMSEA	SRMR
Exploratory Factor Analyses (EFA)								
1-factorEFA LAHCS	746	65	1489.08	22.91	0.57	0.49	0.17	0.13
2-factor EFA LAHCS	746	53	981.04	18.51	0.72	0.59	0.15	0.09
3-factorEFA LAHCS	746	42	679.43	16.18	0.81	0.64	0.14	0.05
4-factor EFA LAHCS	746	32	170.47	5.33	0.96	0.90	0.08	0.02
Confirmatory Factor Analysis (CFA)								
CFA of LAHCS	692	59	264.91	4.49	0.93	0.91	0.07	0.05
CFA of other leadership scales	1442	2238	8193.61	3.66	0.91	0.90	0.04	0.05
Benchmark model(5-factor)	1444	125	903.20	7.23	0.91	0.89	0.07	0.05
Model 1 (4-factor)	1444	129	2599.84	20.15	0.72	0.66	0.12	0.11
Model 2 (4-factor)	1444	129	2666.78	20.67	0.71	0.65	0.12	0.12
Model 3 (4-factor)	1444	129	2793.05	21.65	0.69	0.64	0.12	0.13
Model 4 (4-factor)	1444	129	2504.38	19.41	0.73	0.68	0.11	0.11
Model 5 (1-factor)	1444	135	4648.17	34.43	0.48	0.41	0.15	0.14

*Note*. CFI = comparative fit index; TLI = Tucker–Lewis Index; RMSER = root-mean-square error of approximation. SRMR = standardized root-mean-square residual.

**Table 4:** Mean, standard deviations, correlations, and reliabilities of LAHCS in Study 1.

	*M*	*SD*	*α*	1	2	3	4
1. Exploration	1.74 (1.77)	0.75 (0.75)	0.87 (0.86)		0.46 **	0.55 **	0.09 *
2. Behavioral Change	2.37 (2.42)	1.13 (1.09)	0.90 (0.86)	0.45 **		0.52	−0.14 **
3. Emotional Exchange Awareness	2.10 (2.14)	0.96 (0.98)	0.86 (0.85)	0.54 **	0.59 **		0.04
4. Distance Awareness	2.72 (2.72)	1.27 (1.28)	0.85 (0.81)	0.18 **	−0.01	0.04	

*Note*. The correlation coefficients of the first subsample (*n* = 753) are below the diagonal. The correlation coefficients of the second subsample (*n* = 701) are below the diagonal. * *p* < 0.05; ** *p* < 0.01. The mean, standard deviations, and reliability of the second subsample are listed in parentheses.

**Table 5:** Disattenuated bivariate correlations between LAHCS, GMP and other leadership scales.

Variable	LAHCS			
	Awareness of Holistic Emotional Exchanges	Exploration of Holistic Emotional Exchanges	Awareness of Distance	Includes LAHCS
1. Group Member Prototypicality	0.21 **	0.12 **	0.02	0.26 **
2. Leader Group Prototypicality	0.30 **	0.28 **	−0.27	0.49 **
3. Transformational Leadership	0.48 **	0.35 **	−0.24 **	0.57 **
4. Transactional Leadership	0.46 **	0.35 **	−0.31 **	0.55 **
5. Leader–Member Exchange	0.45 **	0.34 **	−0.32 **	0.56 **
6. Authentic Leader	0.46 **	0.37 **	−0.21 **	0.56 **
7. Servant Leadership	0.46 **	0.39 **	−0.30 **	0.52 **

*Note*. ** *p* < 0.01; Listwise *N* = 1382.

**Table 6:** Means, standard deviations, correlations, and reliabilities of variables in Study 2.

	*M*	*SD*	*α*	1	2	3	4	5	6	7	8
1. Exploration	2.33 (1.78)	0.82 (0.94)	0.84 (0.92)		0.57 **	0.59 **	0.36 **	−0.03	−0.04	0.09	0.21 **
2. Behavioral Change	2.71 (2.45)	0.89 (1.10)	0.79 (0.88)	0.43 **		0.62 **	0.28 **	−0.10	−0.11 *	0.29 **	0.28 **
3. Emotional Exchange Awareness	2.57 (2.43)	1.00 (1.07)	0.86 (0.90)	0.57 **	0.53 **		0.31 **	−0.09	−0.11 *	0.24 **	0.32 **
4. Distance Awareness	2.89 (2.58)	1.07 (1.23)	0.75 (0.76)	0.24 **	0.00	−0.02		0.09	0.02	−0.15 **	0.08
5. Emotional Exhaustion	2.02 (3.58)	1.26 (1.73)	0.86 (0.95)	0.28 **	−0.02	0.20 *	0.22 *		0.72 **	−0.36 **	−0.40 **
6. Cynicism	1.68 (3.29)	1.32 (1.74)	0.86 (0.92)	0.19	−0.10	0.07	0.25 *	0.61 **		−0.38 **	−0.61 **
7. Supervisor Satisfaction	2.37 (2.14)	0.67 (0.87)	0.75 (0.84)	0.00	0.26 **	0.13	−0.42 **	−0.31 **	−0.50 **		0.36 **
8. Affective Commitment	4.81 (4.25)	1.02 (1.34)	0.77 (0.88)	−0.19	0.22 *	0.04	−0.30 **	−0.17	−0.40 **	0.51 **	

*Note*. The correlation coefficients of the Italian sample (*N* = 188) are below the diagonal. The correlation coefficients of the American sample (*N* = 367) are below the diagonal. * *p* < 0.05; ** *p* < 0.01. The mean, standard deviations, and reliability of the American sample are listed in parentheses.

**Table 7:** Results of measurement invariance of the LACHS with the Italian and American samples.

Models (M)	Model Fit					Model Difference			
	χ^2^	*df*	RMSEA	CFI	SRMR	ΔM	ΔCFI	Δ*df*	Δχ^2^
Model_Italy_	71.59	59	0.045	0.97	0.059				
Model_USA_	146.69	59	0.064	0.96	0.041				
M1: Configural	226.07	118	0.062	0.96	0.045				
M2: Metric	242.49	127	0.062	0.96	0.055	M2-M1	0.00	9	16.42
M3: Scalar	250.66	136	0.060	0.96	0.055	M3-M2	0.00	9	8.17
M4: Residual	297.10	149	0.065	0.95	0.065	M4-M3	0.01	13	46.44 **

*Note*. At each step in the sequence of invariance tests, all earlier constraints remain in place. RMSEA = robust root-mean-square error of approximation; CFI = robust comparative fit index; SRMR = robust standardized root-mean-square residual; Δ = difference between the comparison and nested model; ** *p* < 0.01.

**Table 8:** Results of the hierarchical regression analyses on the Italian and American samples.

**Italian Sample**								
**Model**	***R***	***R* ^2^**	**Adjusted *R*^2^**	**Change Statistics**				
				***R*^2^ Change**	***F*** **Change**	***df1***	***df2***	**Sig. *F*** **Change**
Job Burnout								
Step 1	0.259 (a)	0.067	0.036	0.067	2.129	6	177	0.052
Step 2	0.375 (b)	0.140	0.106	0.073	14.934	1	176	0.000
Affective Commitment								
Step 1	0.517 (a)	0.267	0.243	0.267	10.997	6	181	0.000
Step 2	0.585 (b)	0.342	0.316	0.075	20.395	1	180	0.000
Satisfaction with Supervisor								
Step 1	0.496 (a)	0.246	0.221	0.246	9.684	6	178	0.000
Step 2	0.533 (b)	0.256	0.256	0.038	9.354	1	177	0.003
**U.S. Sample**								
**Model**	***R***	***R* ^2^**	**Adjusted *R*^2^**	**Change Statistics**				
				***R*^2^ Change**	***F*** **Change**	***df1***	***df2***	**Sig. *F*** **Change**
Job Burnout								
Step 1	0.451 (a)	0.203	0.189	0.203	14.591	6	343	0.000
Step 2	0.465 (b)	0.216	0.200	0.013	5.532	1	342	0.019
Affective Commitment								
Step 1	0.571 (a)	0.326	0.314	0.326	27.669	6	344	0.000
Step 2	0.572 (b)	0.328	0.314	0.002	1.023	1	343	0.313
Satisfaction with Supervisor								
Step 1	0.694 (a)	0.482	0.473	0.482	54.635	6	352	0.000
Step 2	0.706 (b)	0.499	0.489	0.017	11.594	1	351	0.001

*Note*. (a) Predictors: (Constant), transformational leadership, transactional leadership, leader group prototypicality, leader–member exchange, authentic leadership, and servant leadership. (b) Predictors: (Constant), transformational leadership, transactional leadership, leader group prototypicality, leader–member exchange, authentic leadership, servant leadership, and LAHCS.

## Data Availability

The data of the present study are unavailable as participants did not provide their permission to share raw data.

## Supplementary Materials

The following supporting information can be downloaded at: https://www.mdpi.com/article/10.3390/ejihpe16050061/s1. Table S1: Means, standard deviations, and reliabilities of incremental validity variables in the Italian and American samples.

## Appendix A

Italian and English versions of the Leader Awareness of Holistic Contagion Scale.

**Italian Version**	**English Version**
Leader Awareness of Holistic Contagion Scale	
1. Il mio capo è consapevole che mi sento più in confidenza con i miei colleghi che non con lui/lei	1. My leader is aware that I share more confidences with my colleagues, rather than with her/him
2. Il mio capo esplora con me se le emozioni che sperimento al lavoro sono più associate ai miei colleghi, ai clienti con cui interagisco, ai superiori con cui interagisco, piuttosto che con lui/lei	2. My leader explores with me whether the emotions I feel at work are more associated with my colleagues, my clients, and/or other superiors I deal with, rather than with her/him
3. Il mio capo è consapevole che mi sento affettivamente più distante da lui/lei rispetto ai miei colleghi	3. My leader is aware that I feel emotionally more distant from her/him in comparison to my colleagues
4. Il mio capo approfondisce con me le ragioni per cui mi sento più in confidenza con i miei colleghi, piuttosto che con lui/lei	4. My leader explores with me the reasons why I share more confidences with my colleagues, rather than with her/him
5. Il mio capo esplora con me se mi capita di sperimentare il contagio di emozioni più con i clienti che non con lui/lei	5. My leader explores with me if I happen to experience more emotional contagion with my clients, rather than with her/him
6. Il mio capo è consapevole delle emozioni che io e lui/lei ci contagiamo a vicenda	6. My leader is aware of the emotions that we mutually infect each other with
7. Il mio capo è consapevole delle emozioni che i clienti e lui/lei si contagiano a vicenda	7. My leader is aware of the emotions that he or she and their clients mutually infect each other with
8. Il mio capo è consapevole delle emozioni che lui/lei e i suoi superiori si contagiano a vicenda	8. My leader is aware of the emotions that he or she and their superiors mutually infect each other with
9. Il mio capo approfondisce con noi collaboratori le ragioni per cui ci sentiamo più in confidenza tra noi colleghi, piuttosto che con lui/lei	9. My leader explores with us as followers, the reasons why we share more confidences with each other, rather than with her/him
10. Il mio capo esplora con noi collaboratori se le emozioni che sperimentiamo al lavoro sono più associate ai nostri colleghi, ai clienti con cui interagiamo, ai superiori con cui interagiamo, piuttosto che con lui/lei	10. My leader explores with us as collaborators, whether the emotions that we feel at work are more associated with our colleagues, our clients, and/or other superiors we deal with, rather than with her/him
11. Il mio capo esplora con noi collaboratori quanto ci sentiamo a nostro agio con lui/lei e gli/le confidiamo le nostre emozioni, rispetto a quanto facciamo tra noi colleghi	11. My leader explores with us as collaborators, how comfortable we feel with her/him and share our emotions, as compare to what we do with our colleagues
12. Anche se il mio capo non fosse consapevole degli scambi affettivi che avvengono nel nostro contesto, sarebbe disponibile ad includere la gestione di questo aspetto nei suoi comportamenti di leadership	12. Even if my leader were not aware of the emotional exchanges that occur within our work context, he or she would be willing to include the management of this aspect in their leadership behaviors
13. Anche se il mio capo non fosse consapevole degli scambi affettivi che avvengono nel nostro contesto, sarebbe in grado di includere efficacemente la gestione di questo aspetto nei suoi comportamenti di leadership	13. Even if my leader were not aware of the emotional exchanges that occur within our work context, he or she would be able to efficaciously include the management of this aspect in their leadership behaviors
