# Supplementary material for: Integrating Emotional Contagion into Leadership Theorizing: Development and Validation of the Leader Awareness of Holistic Contagion Scale
Source: Eur J Investig Health Psychol Educ. 2026 Apr 29;16(5):61. doi: 10.3390/ejihpe16050061 (PMC13205571; doi:10.3390/ejihpe16050061)
Supplement: Supplementary file 1 [file ejihpe-16-00061-s001.zip › ejihpe-4207308-supplementary.pdf]

Table S1

Means, Standard Deviations, and Reliabilities of Incremental Validity Variables in the Italian and American Samples.

	N	*M*	*SD*	*α*
1. Burnout	184(350)	1.93(3.44)	1.13(1.61)	.89(.95)
2. Commitment	188(351)	4.64(4.25)	1.07(1.34)	.79(.88)
3. Supervisor Satisfaction	185(359)	2.23(2.12)	.69(.88)	.72(.77)
4. LAHCS Total	188(367)	2.52(2.31)	.69(.82)	.88(.92)
5. Transformational	188(367)	3.21(3.24)	.65(.90)	.91(.96)
6. Transactional	188(367)	3.31 (3.28)	.84(1.07)	.73(.89)
7. LMX	188(367)	3.11 (3.18)	.82(1.05)	.86(.93)
8. Authentic	188(367)	3.01 (3.03)	.69(1.01)	.79(.92)
9. Servant	188(367)	2.94 (2.85)	.70(.99)	.89(.95)
10. LGP	188(367)	3.91(4.04)	1.23(1.31)	.91(.93)

Note: The means, standard deviations, and reliabilities of the American subsample are listed in parentheses. LGP = Leader Group Prototypicality.
